# Anti-*Mycobacterium tuberculosis* Terpenoids from *Resina Commiphora*

**DOI:** 10.3390/molecules24081475

**Published:** 2019-04-15

**Authors:** Chuan-Zhi Zhu, Bin-Yuan Hu, Jia-Wang Liu, Yi Cai, Xin-Chun Chen, Da-Peng Qin, Yong-Xian Cheng, Zong-De Zhang

**Affiliations:** 1 Laboratory of Molecular Biology, Beijing Tuberculosis and Thoracic Tumor Research Institute, Beijing 101149, China; 15910404956@163.com; 2 Guangdong Key Laboratory for Genome Stability & Disease Prevention, School of Pharmaceutical Sciences, School of Basic Medicine, Shenzhen University Health Science Center, Shenzhen 518060, China; HuBinyuan2018@163.com (B.-Y.H.); liujiawang@imm.ac.cn (J.-W.L.); caiyi0113@gmail.com (Y.C.); chenxinchun@szu.edu.cn (X.-C.C.); tqindp@szu.edu.cn (D.-P.Q.)

**Keywords:** *Resina Commiphora*, plant resins, terpenoids, anti-*Mycobacterium tuberculosis*

## Abstract

Four new compounds including two new sesquiterpenoid dimers, commiphoroids **E** (**1**) and **F** (**2**), a new triterpenoid (**3**), and a new sesquiterpenoid (**4**), along with three known terpenoids (**5**−**7**) were isolated from *Resina Commiphora*, whose structures were identified by NMR spectra, HRESIMS, and X-ray diffraction analysis. Compounds **1** and **2** both bear an *O*-bridge ring and feature a plausible [4 + 2] Diels–Alder cycloaddition reaction. Antimycobacterial activities show that all the tested compounds (200 μM) could inhibit the growth of both sensitive and clinically multi-drug resistant (MDR) isolated strains. In addition, cellular toxicity of the isolates against human cancer cells and THP-1 monocyte cells was examined.

## 1. Introduction

**Figure 1:**
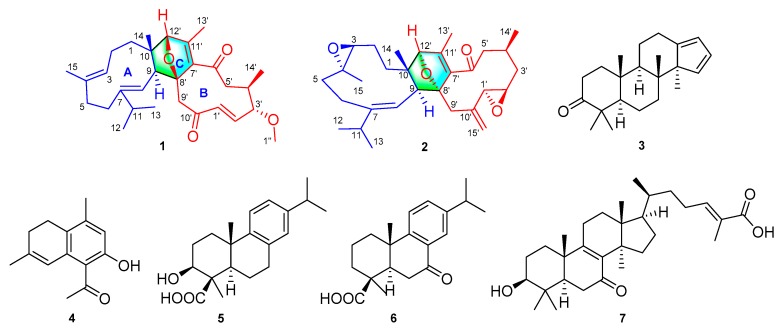
The structures of compounds **1**–**7**.

Tuberculosis (TB) is one of the leading causes of human mortality by a single infectious agent *Mycobacterial tuberculosis* (*M. tuberculosis*). This is illustrated by the approximately 10.0 million infected people and 1.3 million deaths worldwide in 2017 [1]. Drug resistance, including multi-drug resistant (MDR), extensively drug-resistant (XDR), and totally drug resistant (TDR), has been spreading worldwide, particularly in India, China, and Russia [1]. Because of the lack of effective new anti-tuberculosis drugs, TB treatment remains challenging. Development of novel agents that could effectively fight against *M. tuberculosis* are thus of great importance. Within this context, search for anti-*Mycobacterium tuberculosis* agents have received attention in recent years and increasing numbers of active compounds have been isolated from natural origins or synthesized [2,3]. Aromatic plants have been used for treating infective diseases, including air cleaning in China since ancient times. In fact, aromatic therapy is also popular worldwide, which inspired our search for anti-TB agents from aromatic plants. The resins from the bark of *Commiphora* plants, also known as myrrh, have been historically used as aromatic resins in ancient Egypt for wound healing and embalming, which implies that myrrh might contain antimicrobial agents. With this in mind and as a part of our continuous efforts on myrrh [4,5], this study afforded two new sesquiterpenoid dimers (**1** and **2**), a new triterpene (**3**), a new sesquiterpenoid (**4**), and three known terpenoids (**5**−**7**) [6,7,8] (Figure 1). Their antimicrobial properties against multiple *M. tuberculosis* strains including sensitive and MDR were observed. 

## 2. Results and Discussion

### 2.1. Structure Elucidation of the Compounds

Commiphoroid **E** (**1**), obtained as colorless needle crystals (MeOH), has a molecular formula of C_30_H_42_O_4_ (ten degrees of unsaturation) on the basis of its HRESIMS (*m/z* 467.3150, calculated 467.3156 [M + H]^+^), ^13^C NMR, and DEPT spectra (Supplementary Materials). The ^1^H NMR data (Table 1) of **1** exhibit six methyl (3 singlets and 3 doublets), one methoxy group (*δ*_H_ 3.21), and four olefinic methine protons. The ^13^C NMR and DEPT spectra display 30 carbon resonances including six methyl, one methoxy, six methylene, nine methine (four olefinic, two oxygenated, and three aliphatic), and eight non-protonated carbons (two keto-carbonyls, four olefinic, two aliphatic including one oxygenated). In consideration of the aforementioned data and the chemical profile of the genus *Commiphora*, we speculated that **1** might be a sesquiterpenoid dimer.

**Figure 2:**
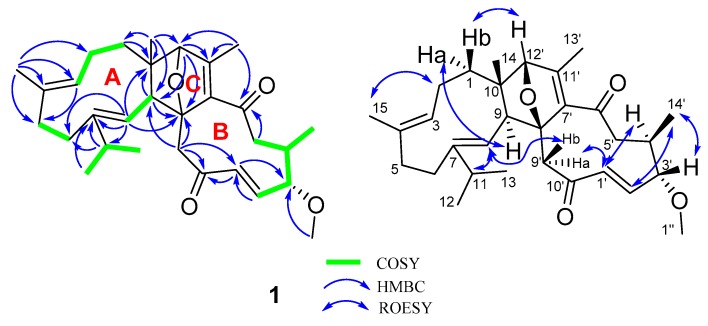
Key ^1^H-^1^H COSY and HMBC correlations for **1**.

The structural architecture of **1** was mainly carried out by using 2D NMR data. The ^1^H−^1^H COSY spectrum of **1** shows correlations: H_2_-1/H_2_-2/H-3 (*δ*_H_ 4.83), H-5/H-6, H-8(*δ*_H_ 5.05)/H-9, H_3_-12/H-11/H_3_-13, and H-2′ (*δ*_H_ 5.95)/H-3′/H-4′/H_2_-5′/H_3_-14′ (Figure 2). Based on the observed HMBC correlations of H_3_-15/C-2, C-3 (*δ*_C_ 122.3), C-4 (*δ*_C_ 134.5), C-5, C-6, H-11/C-6, C-7 (*δ*_C_ 146.7), C-8 (*δ*_C_ 121.1), H-8 (*δ*_H_ 5.05)/C-6, C-7 (*δ*_C_ 146.7), C-9, C-10, and H_3_-14/C-9, C-10, C-1, we established the structure of part A in **1** (Figure 2). Inspection of the remaining carbon signals in the ^13^C NMR spectrum of **1** found that they are similar to those of myrrhterpenoid E [9]. The difference between them is a *Δ*^10′(15′)^ exocyclic double bond and an ester carbonyl at C-12′ are replaced by a keto-carbonyl and a methine group. These alterations are supported by the HMBC correlations of H-1′ (*δ*_H_ 5.96), H-9′/C-10′ (*δ*_C_ 202.2) and H_3_-13′/C-12′ (*δ*_C_ 91.7). The structure of part B in **1** was therefore identified. The notion that parts A and B are connected via C-9−C-8′ and C-10−C-12′ is confirmed by HMBC correlations of H-8, H-9/C-8′, H-9′, H-12′/C-9, H-12′/C-10, and H_3_-14/C-12′. Collectively, the planar architecture of **1** was deduced.

**Figure 3:**
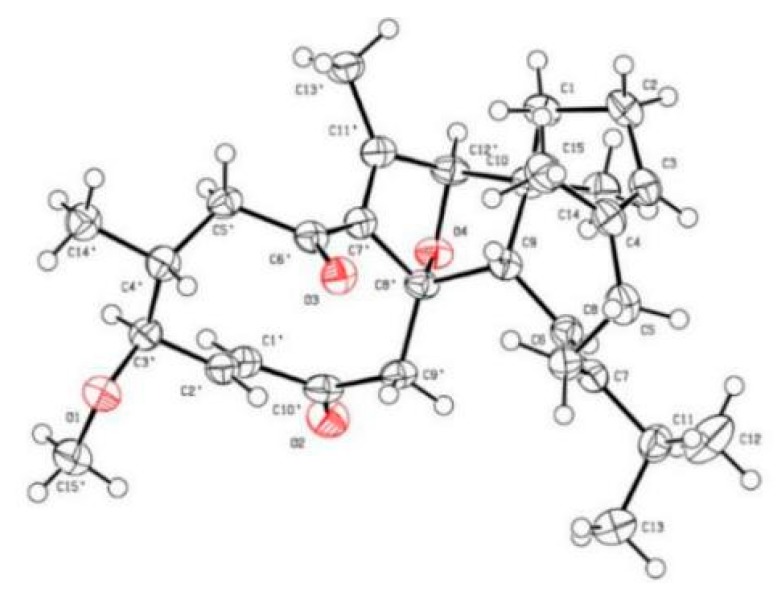
X-ray crystallographic structure of **1**.

It is evident that the presence of an oxygen bridge in **1** makes a rigid C ring, naturally allowing the assignment of the relative configurations at C-8′ and C-12′. Additionally, ROESY correlations of H-9/Ha-1 and Hb-1/H-12′ are observed, indicating the stereochemistry at C-9 and C-10. As for the relative configurations at the chiral centers of the B ring, the observed ROESY correlations of H_3_-14′/H-2′, H-3′ and H-1′/H-4′ indicate their spatial vicinity. In addition, ROESY correlations of H-8/H-11, H-2/H_3_-15, and H-1′/H-4′ indicate that the *Δ*^7(8)^, *Δ*^3′(4′)^, and *Δ*^1′(2′)^ double bonds are all *E-*oriented. To finally clarify the absolute configuration of **1**, a crystal was fortunately afforded, a subsequent single-crystal X-ray diffraction analysis with CuKα radiation (Figure 3) allowed us to assign the absolute configuration of **1** as 9*S*,10*S*, 3′*S*,4′*R*,8′*R*,12′*R*.

**Figure 4:**
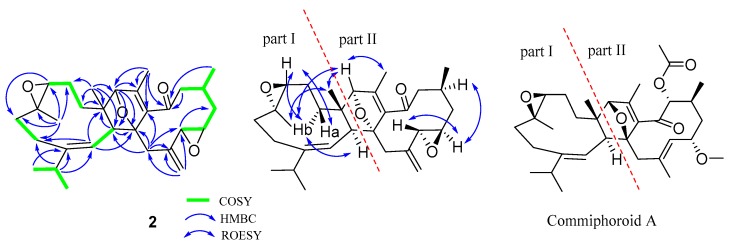
Key ^1^H-^1^H COSY and HMBC correlations for **2** and the structure of commiphoroid A.

Commiphoroid **F** (**2**), obtained as colorless needle crystals (MeOH), was found to possess a molecular formula of C_30_H_42_O_4_ (ten degrees of unsaturation) derived from its HRESIMS (*m/z* 489.2917, calculated 489.2981 [M + Na] ^+^), ^13^C NMR, and DEPT spectra (Supplementary Materials). The ^1^H NMR data (Table 1) of **2** exhibit six methyl (three singlets and three doublets) and three olefinic methine protons (*δ*_H_ 4.44, 5.07, and 4.88). The ^13^C NMR and DEPT spectra display 30 carbons ascribed to six methyl, eight methylene (one olefinic), eight methine (one olefinic, four oxygenated, and three aliphatic), and eight non-protonated carbons (one keto-carbonyl, four olefinic, three aliphatic including two oxygenated). These data indicate that **2** might be a sesquiterpenoid dimer. Inspection of NMR data found that the NMR signals of part I in **2** resemble those of part I in commiphoroid A [10]. The remaining NMR data of **2** are similar to those of 1,2-epoxyfuruno-10(15)-germacren-6-one [11], differing in that a furan ring in it is replaced by a 2,5-dihydrofuran in **2**. This change is supported by the HMBC correlations of H_3_-13′/C-12′ (*δ*_C_ 92.3), H-9′/C-8′ (*δ*_C_ 94.5), C-7′ (*δ*_C_ 140.3). As shown in Figure 4, parts I and II of 1 are fused via C-9−C-8′ and C-10−C-12′ supported by HMBC correlations of H-9/C-8′, H-8/C-8′, H-9′/C-9, H-12′/C-9, C-10, and H_3_-14/C-12′. In this way, the planar structure of **2** was identified.

**Figure 5:**
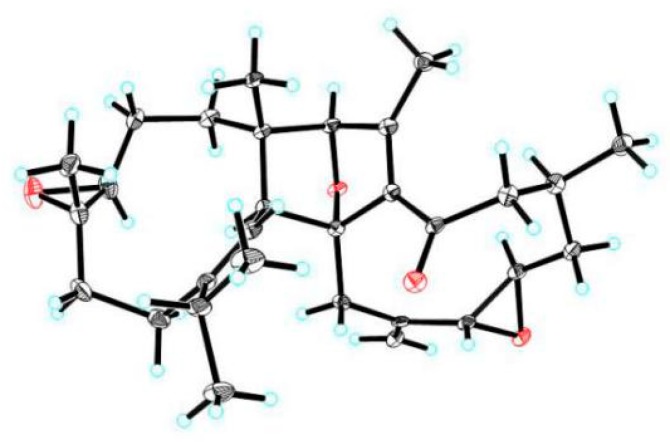
X-ray crystallographic structure of **2**.

The relative configurations at part I of **2** are the same as those of commiphoroid A by analysis of its ROESY correlations (Figure 4). As for the stereochemistry at part II, ROESY correlations of H-4′/H-2′ and H-12′/H_3_-13′ are observed, indicating their spatial relationship. In addition, a ROESY correlation of H-8/H-11 indicates that the *Δ*^7(8)^ double bond is *E*-configurated. However, it is hard to clarify the stereochemistry at the chiral centers of the macro ring due to the flexible conformers. The stereochemistry of **2** was finally assigned as 3*S*,4*S*,9*S*,10*S*,1′*R*,2′*R*,4′*S*,8′*R*,12′*S* by a single-crystal X-ray diffraction analysis (Figure 5).

**Figure 6:**
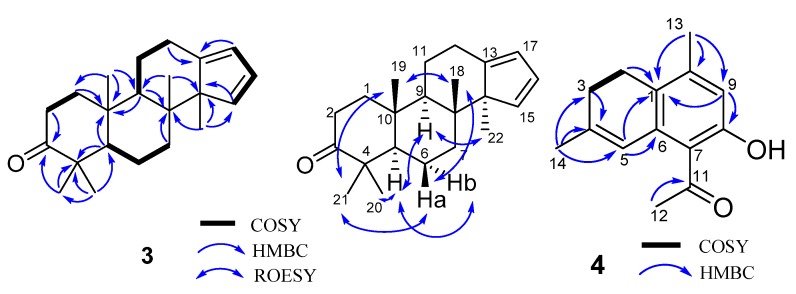
Key ^1^H-^1^H COSY and HMBC correlations for **3** and **4**.

Compound **3**, obtained as a white powder, was found to possess a molecular formula of C_22_H_32_O (seven degrees of unsaturation) derived from its HRESIMS (*m/z* 312.2449, calcd 312.2453 [M]^+^), ^13^C NMR, and DEPT spectra (Supplementary Materials). The ^1^H NMR spectrum (Table 2) of **3** exhibits five methyl (five singlets) and three sp^2^ methine (*δ*_H_ 6.17, 6.23, and 5.82). The ^13^C NMR and DEPT spectra display 22 carbons ascribed to five methyl, six methylene, five methine (three sp^2^ ones), and six non-protonated carbons (one keto-carbonyl, one olefinic and four aliphatic). These data indicate that **3** might be a triterpenoid. Inspection of its NMR data found that the NMR signals of **3** are similar to those of commiphorane G2 [12]. The difference between them is a hydroxy group at C-3 in commiphorane G2 is replaced by a keto-carbonyl in **3**. This alteration is supported by the HMBC correlations (Figure 6) of H-2/C-3 and H_3_-21/C-3. Thus, the planar structure of **3** was identified and named as 3-oxo-commiphorane G2. For the stereochemistry of **3**, ROESY correlations of Ha-6/H_3_-21, H_3_-18; H_3_-19/H_3_-21, H_3_-18; H-9/H-22, H-5, and H-5/Hb-6, H_3_-20 clearly indicate the stereochemistry at chiral centers to be 5R*,8R*,9R*,10R*,14R*.

Compound **4**, obtained as a brown oil, possesses a molecular formula of C_14_H_16_O_2_ (seven degrees of unsaturation) derived from its HRESIMS (*m/z* 217.1219, calculated 217.1223 [M + H]^+^), ^13^C NMR, and DEPT spectra (Supplementary Materials). The ^1^H NMR spectrum (Table 2) of **4** exhibits three methyl (three singlets) and two sp^2^ methine (*δ*_H_ 6.42, 6.61). The ^13^C NMR and DEPT spectra display 14 carbons classified into three methyl, two methylene, two methine (two olefinic), and seven non-protonated carbons (one keto-carbonyl and six olefinic). These data indicate that **4** is a norsesquiterpenoid resembling (4*α*)-8-hydroxy-12-norcardina-6,8,10-trien-11-one [13]. The difference is that a *Δ*^4(5)^ double bond appears in the framework of **4**, which gains supports from HMBC evidences (Figure 6) of H_3_-14/C-4, C-5, and H-3/C-4. As a result, the structure of **4** was deduced to be 8-hydroxy-12-norcardina-4,6,8,10-tetraen-11-one.

The known compounds were identified as nepetaefolin F (**5**) [6], 7-oxocallitrisic acid (**6**) [7], 7-oxo-ganoderic acid Z (**7**) [8], respectively, by comparison of their spectroscopic data with those reported in the literature.

### 2.2. Biological Evaluation

It is well known that dasetherapy has been used for the treatment of pulmonary tuberculosis, and forest aromatic substances such as terpenes were considered beneficial for patients suffering from pulmonary tuberculosis [14]. In this study, we proposed that aromatic plants might be a potential source of active agents against *M. tuberculosis*. With this, compounds **1** and **5**−**7** were evaluated for their antimycobacterial activities against sensitive strains (H37Ra, H37Rv) and clinical MDR isolates (C-200-7, C-200-29, and C-200-39). All the strains were exposed to the same concentration (200 μM) for seven days, and the inhibition of bacterial growth was determined by measuring the absorbance and comparing it with a negative control by REMA assays. The results (Table 3) show that all of the tested compounds and isoniazid (INH) could significantly inhibit the growth of sensitive strains. For the clinical MDR strains, INH was used to confirm three clinical MDR strains that were resistant to INH by clinical microbiological testing. Results showed that all three clinical MDR strains were resistant to INH, compared to H37Ra and H37Rv. Among the clinical strains, the inhibitory potency of the compounds varies in different strains. In comparison, the inhibitory effects of compounds **5** and **6** in clinical MDR strains are better than those of **1** and **7**. In this study, despite the fact that the inhibitory concentration of the isolated compounds against TB is higher than that of INH, it still implies that such structure templates might be helpful for future drug optimization in the field of TB. Last but not the least, the present findings indicate that natural compounds from aromatic plants might possess different mechanisms against TB in contrast to synthesized chemicals such as INH, due to the observations that the former mainly demonstrates inhibition instead of killing toward *M. tuberculosis* strains.

In this study, the cytotoxicity of compounds **1** and **5**−**7** was also determined using human monocyte THP-1 and lung cancer cell line (A549) to get an insight into the cellular toxicity of the isolates. All cells were exposed to the same concentrations (200 μM) for 24 h, and cell viability was quantified by WST-1 assay (Table 4). The results show that compounds **1**, **6**, and **7** are weakly toxic towards THP-1 at 200 μM, whereas this is not the case for compound **5**. Additionally, compounds **5** and **7** exhibit no inhibitory effect on A549 cells even at 200 μM. In contrast, compound **1** (200 μM) was found to be weakly active against human cancer cells (A549).

## 3. Experimental Section

### 3.1. General Procedures

Optical rotations were determined on a Jasco P-1020 polarimeter. UV spectra were recorded on a Shimadzu UV-2401PC spectrometer. CD spectra were obtained on a Chirascan instrument. NMR spectra were measured on a Bruker AV-400 or an AV-800 spectrometer, with TMS as an internal standard. ESIMS and HRESIMS were measured on an API QSTAR Pulsar 1 spectrometer. Silica gel (200–300 mesh; Qingdao Marine Chemical Inc., China), YMC-Pack ODS-A 250 mm × 9.4 mm, i.d., 5 µm, Thermo Hypersil GOLD-C18 250 mm × 21.2 mm, i.d., 5 µm., MCI gel CHP 20P (75–150 μm, Mitsubishi Chemical Industries, Tokyo, Japan), C-18 silica gel (40–60 µm; Daiso Co., Japan) and Sephadex LH-20 (Amersham Pharmacia, Sweden) were used for column chromatography. Semi-preparative HPLC was carried out using an Agilent 1200 liquid chromatograph equipped with an Agilent Zorbax SB-C_18_ column (250 mm × 9.4 mm, i.d., 5 μm).

### 3.2. Plant Resins

The medicinal materials of *Resina Commiphora* (myrrh) were obtained from Juhuacun Market of Material Medica, Kunming, Yunnan Province, PR China, in July 2013. The material was identified by Mr. Bin Qiu at Yunnan Institute of Materia Medica, and a voucher specimen (CHYX-0585-2) was deposited at the School of Pharmaceutical Sciences, Shenzhen University, China in November 2017.

### 3.3. Extraction and Isolation

The dried myrrh (50 kg) were ground and soaked with 95% EtOH (180 L, 3 × 48 h) to give a crude extract, which was suspended in warm water followed by extraction with EtOAc to afford an EtOAc soluble extract (8.0 kg). This extract was divided into six parts (Fr.A–Fr.F) using a silica gel column chromatography eluted with petroleum ether–acetone (100:0, 100:1, 60:1, 40:1, 20:1, 5:1, 3:1, 1:1, 0:100). Fr.B (2.4 kg) was further separated via a silica gel column washed with petroleum ether–EtOAc (100:0, 100:1, 60:1, 40:1, 20:1, 5:1, 3:1, 1:1) and petroleum ether–acetone (5:1, 3:1, 1:1) to provide six portions (Fr.B.1–Fr.B.6). Fr.B.1 (240 g) was subjected to an MCI gel CHP 20P column washed with gradient aqueous MeOH (30–100%) to provide seven portions (Fr.B.1.1–Fr.B.1.7). Fr.B.1.3 (13.0 g) was separated via C-18 eluted with aqueous MeOH (40–100%) to provide three portions (Fr.B.1.3.1–Fr.B.1.3.3). Fr.B.1.3.2 (7.5 g) was divided into three portions (Fr.B.1.3.2.1–Fr.B.1.3.2.3) by C-18 eluted with aqueous MeOH (55–100%). Among them, Fr.B.1.3.2.2 (1.1 g) was submitted to Sephadex LH-20 (MeOH) followed by semi-preparative HPLC (aqueous MeOH, 80%) to give **4** (1.2 mg, *t*_R_ = 11.3 min; flow rate: 3 mL/min). Fr.B.5 (186.6 g) was separated via MCI gel CHP 20P eluted with aqueous MeOH (55–100%) to provide eight portions (Fr.B.5.1–Fr.B.5.8). Fr.B.5.5 (16 g) was separated via C-18 eluted with aqueous MeOH (50–100%) to provide nine portions (Fr.B.5.5.1–Fr.B.5.5.9). Fr.B.5.5.7 (4.0 g) was submitted to Sephadex LH-20 (MeOH) to yield five fractions (Fr.B.5.5.7.1–Fr.B.5.5.7.5). Further semi-preparative HPLC separation on Fr.B.5.5.7.2 (21.2 mg) by aqueous MeCN (70%) afforded Fr.B.5.5.7.2.8 (10.6 mg), which was purified by semi-preparative HPLC (aqueous MeOH, 80%) to give **2** (0.9 mg, *t*_R_ = 19.9 min; flow rate: 3 mL/min). Fr.B.5.7 (17.0 g) was subjected to a C-18 column washed with aqueous MeOH (40–100%) to provide fourteen portions (Fr.B.5.7.1–Fr.B.5.7.14). Fr.B.5.7.5 (510.7 mg) was submitted to Sephadex LH-20 (MeOH) to yield three fractions (Fr.B.5.7.5.1−Fr.B.5.7.5.3). Fr.B.5.7.5.3 (20.0 mg) was purified by semi-preparative HPLC (aqueous MeCN, 55%) to give two portions. Compound **7** (0.8 mg, *t*_R_ = 12.9 min; flow rate: 3 mL/min) was purified from Fr.B.5.7.5.3.2 (2.1 mg)) by HPLC separation (aqueous MeOH, 78%). Fr.B.5.7.10 (1.98 g) was subjected to a MCI gel CHP 20P column washed with gradient aqueous MeOH (60–100%) to provide fourteen portions (Fr.B.5.7.10.1–Fr.B.5.7.10.14). Fr.B.5.7.10.2 (320.0 mg) was divided into ten parts (Fr.B.5.7.10.2.1–Fr.B.5.7.10.2.10) using a vacuum liquid chromatography on silica gel eluted with petroleum ether–acetone (50:0, 30:1, 20:1, 10:1, 8:1, 6:1, 3:1, 2:1, 1:1). Further semi-preparative HPLC on Fr.B.5.7.10.2.5 (35.1 mg) by aqueous MeOH (86%) afforded Fr.B.5.7.10.2.5.3 (11.3 mg), then it was purified by semi-preparative HPLC (aqueous MeCN, 83%) to give **1** (1.8 mg, *t*_R_ = 14.9 min; flow rate: 3 mL/min). Fr.B.5.8 (25.8 g) was separated via a C-18 column eluted with aqueous MeOH (50–100%) to provide ten portions (Fr.B.5.8.1–Fr.B.5.8.10). Fr.B.5.8.6 (0.6 g) was submitted to Sephadex LH-20 (MeOH) to yield five fractions (Fr.B.5.8.6.1–Fr.B.5.8.6.5). Compounds **5** (2.1 mg, *t*_R_ = 14.0 min; flow rate: 3 mL/min) and **6** (1.7 mg, *t*_R_ = 17.1 min; flow rate: 3 mL/min) was afforded from Fr.B.5.7.5.3.2 (23.2 mg) by HPLC separation (aqueous MeOH, 62%). Fr.B.5.8.9 (4.7 g) was submitted to Sephadex LH-20 (MeOH) to yield seven fractions (Fr.B.5.8.9.1–Fr.B.5.8.9.7). Of which Fr.B.5.8.9.6 (160.0 mg) was fractionated by semi-preparative HPLC (aqueous MeOH, 90%) to give **3** (1.7 mg, *t*_R_ = 26.6 min; flow rate: 3 mL/min).

### 3.4. Compound Characterization Data

Commiphoroid **E** (**1**): colorless needle crystals (MeOH), [*α*]_D_^25^ +8.8 (*c* 0.04, MeOH); UV (MeOH) *λ*_max_ (log *ε*) 226 (0.43, sh); ESIMS (positive) *m/z* 467 [M + H]^+^; HRESIMS *m/z* 467.3150 [M + H]^+^ (calculated for C_30_H_43_O_4_, 467.3156); ^1^H and ^13^C NMR data, see Table 1.

Commiphoroid **F** (**2**): colorless needle crystals (MeOH); [*α*]_D_^25^ + 26.58 (*c* 0.16, MeOH); UV (MeOH) *λ*_max_ (log *ε*) 248 (3.72), 203 (4.05); ESIMS (positive) *m/z* 489 [M + Na] ^+^; HRESIMS *m/z* 489.2974 [M + Na]^+^ (calculated for C_30_H_42_NaO_4_ 489.2981); ^1^H and ^13^C NMR data, see Table 1.

3-Oxo-commiphorane G2 (**3**): white powders; [α]_D_^23^ +11.3 (*c* 0.13, MeOH); EIMS (positive) *m/z* 312 [M]^+^; HREIMS *m/z* 312.2449 [M]^+^ (calculated for C_22_H_32_O 312.2453); ^1^H and ^13^C NMR data, see Table 2.

8-Hydroxy-12-norcardina-4,6,8,10-tetraen-11-one (**4**): brown oils; ESIMS (positive) *m/z* 217 [M + H]^+^; HRESIMS *m/z* 217.1219 [M + H]^+^ (calculated for C_14_H_1__7_O_2_ 217.1223); ^1^H and ^13^C NMR data, see Table 2.

### 3.5. X-ray Crystallographic Analysis of ***1*** and ***2***

Crystal data for compound **1**: Data were collected using a Sapphire CCD with a graphite monochromated Cu K*α* radiation, *λ* = 1.54184 Å at 100 K. Crystal data: C_30_H_42_O_4_, *M* = 466.63 g/mol, space group *P*2_1_2_1_2_1_; unit cell dimensions were determined to be a = 9.7285(3) Å, b = 15.5353(5) Å, c = 17.4067(6) Å, *α* = 90°, *β* = 90°, *γ* = 90°, V = 2630.75(15) Å^3^, Z = 4, T = 100.00(10) K, *Dcalc* = 1.178 g/cm^3^, F (000) = 1016.0, μ (Cu K*α*) = 0.599 mm^−1^, 13,183 reflections measured (7.628° ≤ 2Θ ≤ 147.196°), 5203 unique (*R*_int_ = 0.0435, R_sigma_ = 0.0358), which were used in all calculations. The final *R*_1_ was 0.0575 (I > 2σ(I)) and *wR*_2_ was 0.1596. The final refinement gave *R* = 0.0575(4971), *Rw* = 0.1596(5203), *S* = 1.054, and Flack = 0.04(14). Crystallographic data for structure **1** has been deposited at the Cambridge Crystallographic Data Centre (CCDC 1900483).

Crystal data for compound **2**: Crystal data for cu_q58d_0m: C_30_H_42_O_4_·H_2_O, *M* = 484.65, *a* = 10.9521(4) Å, *b* = 8.4631(4) Å, *c* = 15.3423(7) Å, *α* = 90°, *β* = 106.964(2)°, *γ* = 90°, *V* = 1360.18(10) Å^3^, *T* = 100(2) K, space group *P*21, *Z* = 2, *μ*(CuKα) = 0.624 mm^−1^, 14,901 reflections measured, 4836 independent reflections (*R_int_* = 0.0389). The final *R_1_* values were 0.0430 (*I* > 2*σ*(*I*)). The final *wR*(*F*^2^) values were 0.1122 (*I* > 2*σ*(*I*)). The final *R_1_* values were 0.0455 (all data). The final *wR*(*F*^2^) values were 0.1141 (all data). The goodness of fit on *F*^2^ was 1.062. Flack parameter = 0.05(10). Crystallographic data for structure **1** has been deposited at the Cambridge Crystallographic Data Centre (CCDC 1900642).

The structure was solved by direct methods using the SHELXS-97 program and refined by the program SHELXL-97 and full-matrix least squares calculations. In the structure refinements, non-hydrogen atoms were placed on the geometrically ideal positions by the “ride on” method. Hydrogen atoms bonded to oxygen were located by the structure factors with isotropic temperature factors.

### 3.6. Antibacterial Evaluation against M. tuberculosis

*M. tuberculosis* strains: Attenuated *M. tuberculosis* strain H37Ra, wild-type reference strain H37Rv, and three clinical MDR strains (C-200-7, C-200-29, C-200-39) were used in the modified resazurin microtiter plate-based assay [15]. C-200-7 is resistant to INH, rifampicin, and streptomycin. C-200-29 and C-200-39 are both resistant to INH, rifampicin, ethambutal, and streptomycin. All *M. tuberculosis* strains were cultured in in Difco^TM^ Middlebrook 7H9 (Becton Dickinson, BD, Franklin Lakes, NJ, USA) broth with 10% oleic acid–albumin-dextrose–catalase (OADC) enrichment (BD, Franklin Lakes, NJ, USA), 0.05% (*v*/*v*) Tween 80, and 0.2% (*v*/*v*) glycerol at 37 °C. To determine the effects of compounds bactericidal activity against *M. tuberculosis*, bacterial cultures at mid-log phase were diluted in Middlebrook 7H9 broth with OADC to an absorbance at 600 nm (OD600) of 0.02 using Epoch™ 2 microplate spectrophotometer (BioTek, Winooski, VT, USA). The culture and drugs (200 μM) were transferred to 96-well plates (Corning, Corning, NY, USA), and each 96-well screening plate contained three control columns with 0.5% dimethyl sulfoxide (DMSO). Bacterial cultures were incubated at 37°C with slow shaking horizontally. The bactericidal activity of the compounds was measured from duplicate wells in the absorbance assays after 7-day treatment using Epoch™ 2 microplate spectrophotometer.

### 3.7. Cell Viability Assay

THP-1 monocyte cells and human non-small cell lung cancer cells A549 were obtained from the Cell Bank of China Science Academy (Shanghai, China). The THP-1 was cultured in Roswell Park Memorial Institute (RMPI-1640) and A549 was maintained in Dulbecco′s modified Eagle′s medium (DMEM) and supplemented with 10% fetal bovine serum incubated at 37 °C in an atmosphere of 5% CO_2_. The cell lines were exposed to the same concentrations (200 μM) of compounds **1** and **5**−**7** for 24 h, and DMSO was used as a negative control. The cell viability was measured using WST-1 reagent kit (Beyotime Biotechnology, Shanghai, China). The WST-1 solution was added and incubated for 2–4 h at 37 °C. The absorbance was measured at 450 nm and 650 nm using Epoch™ 2 microplate spectrophotometer (BioTek, Winooski, VT, USA). The cell viability was calculated according to the manufacturer′s instructions.

## 4. Conclusions

In the present study, two new sesquiterpenoid dimers, commiphoroids **E** (**1**) and **F** (**2**), and three known terpenoids (**3**−**5**), were characterized from *Resina Commiphora*, with **1** as a dimeric norsesquiterpenoid. Antimicrobial activities against *M. tuberculosis* of the isolates from aromatic resins not only indicate the scientific rationale of the utilization of aromatic material in antimicrobial field but suggest a new structure scaffold for anti-TB drug development.

## Figures and Tables

**Table 1:** ^1^H (800 MHz) and ^13^C NMR (200 MHz) Data of **1** and **2** in CDCl_3_ (*δ* in ppm, *J* in Hz).

	1			2	
no.	*δ*_H_	*δ*_C_	no.	*δ*_H_	*δ*_C_
**1**	Ha: 1.44, ddd (12.9, 4.7, 1.9)	35.6, CH_2_	**1**	Ha: 2.18, brdd (14.4, 5.9)	41.2, CH_2_
	Hb: 0.99, brs			Hb: 1.78, ddd (14.4, 4.8, 2.9)	
**2**	Ha: 2.15, m 2.68, dd (6.5, 16.9)	23.5, CH_2_	**2**	Ha: 2.03, m	24.2, CH_2_
	Hb: 1.96, m			Hb: 1.47, m	
**3**	4.83, brd (7.7)	122.3, CH	**3**	3.07, brd (10.0)	62.2 *^a^*, CH
**4**		134.5, C	**4**		62.3 *^a^*, C
**5**	Ha: 2.11, brd (12.6)	37.2, CH_2_	**5**	Ha: 2.10, brdd (13.6, 5.1)	36.6, CH_2_
	Hb: 1.77, brdd (12.6, 2.3)			Hb: 1.53, brd (13.6)	
**6**	Ha: 2.30, brd (12.4)	27.4, CH_2_	**6**	Ha: 2.67, brd (12.0)	25.7, CH_2_
	Hb: 2.20, brd (12.4)			Hb: 2.04, m	
**7**		146.7, C	**7**		146.6, C
**8**	5.05, d (11.6)	121.1, CH	**8**	4.44, d (12.0)	121.2, CH
**9**	2.24, d (11.6)	49.5, CH	**9**	2.67, d (12.0)	52.8, CH
**10**		47.6, C	**10**		47.2, C
**11**	2.25, m	35.1, CH	**11**	2.23, m	33.0, CH
**12**	1.05, d (6.7)	23.5, CH_3_	**12**	1.01, d (6.7)	20.9, CH_3_
**13**	1.08, d (6.7)	22.2, CH_3_	**13**	1.12, d (6.7)	22.5, CH_3_
**14**	1.04, s	21.9, CH_3_	**14**	0.73, s	17.2, CH_3_
**15**	1.49, s	17.8, CH_3_	**15**	1.12, s	20.9, CH_3_
**1′**	5.96, d (16.6)	146.2, CH	**1′**	2.74, brs	61.4, CH
**2′**	5.95, dd (16.6, 9.2)	132.1, CH	**2′**	2.25, brdd (10.4, 2.2)	62.2 *^a^*, CH
**3′**	3.18, brd (9.2)	88.4, CH	**3′**	Ha: 2.14, m	40.0, CH_2_
				Hb: 1.02, m	
**4′**	2.67, m	38.4, CH	**4′**	2.17, m	29.6, CH
**5′**	Ha: 2.48 brd (12.1)	47.6, CH_2_	**5′**	Ha: 2.92, brd (14.0)	54.2, CH_2_
	Hb: 2.27 brd (12.1)			Hb: 2.21, m	
**6′**		204.6, C	**6′**		199.5, C
**7′**		147.2, C	**7′**		140.3, C
**8′**		92.9, C	**8′**		94.5, C
**9′**	Ha: 3.31, d (12.9)	41.6, CH_2_	**9′**	Ha: 3.37 d (14.1)	37.1, CH_2_
	Hb: 2.55, d (12.9)			Hb: 2.44 d (14.1)	
**10′**		202.2, C	**10′**		141.9, C
**11′**		146.1, C	**11′**		154.4, C
**12′**	4.07, s	91.7, CH	**12′**	4.13, s	92.3, CH
**13′**	1.99, s	14.9, CH_3_	**13′**	2.19, s	16.4, CH_3_
**14′**	1.17, d (6.8)	18.7, CH_3_	**14′**	1.10, d (6.6)	23.8, CH_3_
**3′-OMe**	3.21, s	57.4, CH_3_	**15′**	Ha: 5.07, brs	111.6, CH_2_
				Hb: 4.88, brs	

*^a^* The symbol in the same column might be interchangeable.

**Table 2:** ^1^H (800 MHz) and ^13^C NMR (200 MHz) Data of **3** and **4** in CDCl_3_ (*δ* in ppm, *J* in Hz).

	3		4	
no.	*δ*_H_	*δ*_C_	*δ*_H_	*δ*_C_
**1**	2.50, m	34.1, CH_2_		125.5, C
	2.49, m			
**2**	2.01, m	40.2, CH_2_	Ha:2.61 overlap	23.8, CH_2_
	1.58, m		Hb:2.22 overlap	
**3**		218.0, C	2.60 overlap	28.0, CH_2_
			2.23 overlap	
**4**		47.3, C		140.5, C
**5**	1.50, m	55.2, CH	6.42 d (1.28)	122.0, CH
**6**	1.50, m	19.7, CH_2_		135.5, C
	1.49, m			
**7**	Ha:1.81, m	36.5, CH_2_		117.8, C
	Hb:1.57, overlap			
**8**		40.9, C		158.8, C
**9**	1.48, overlap	50.4, CH	6.61 s	116.6, CH
**10**		37.3, C		143.0, C
**11**	Ha:1.66, m	23.6, CH_2_		205.6, C
	Hb:1.40, overlap			
**12**	Ha:2.64, m	26.3, CH_2_	2.58 s	32.3, CH_3_
	Hb:2.17, m			
**13**		156.9, C	2.25 s	20.4, CH_3_
**14**		61.0, C	2.01 s	24.1, CH_3_
**15**	6.17, d (5.3)	142.3, CH		
**16**	6.23, dd (5.3, 1.8)	129.7, CH		
**17**	5.82, d (1.8)	120.5, CH		
**18**	0.66, s	15.2, CH_3_		
**19**	0.91, s	16.3, CH_3_		
**20**	1.04, s	21.0, CH_3_		
**21**	1.12, s	27.0, CH_3_		
**22**	1.03, s	16.9, CH_3_		

**Table 3:** The inhibitory activities of the compounds against sensitive and clinically isolated MDR strains.

Compound	Inhibition (200 μM) (%)				
	H37Ra	H37Rv	C-200-7	C-200-29	C-200-39
**1**	21.61 ± 3.18	70.20 ± 6.43	32.83 ± 4.29	41.28 ± 26.79	3.10 ± 4.38
**5**	65.82 ± 5.23	85.86 ± 12.86	58.08 ± 2.14	74.11 ± 6.44	35.48 ± 3.7
**6**	52.58 ± 4.36	85.35 ± 2.86	44.19 ± 2.5	75.78 ± 4.4	38.81 ± 4.38
**7**	95.80 ± 6.88	98.23 ±1.79	32.58 ± 3.21	42.67 ± 17.28	9.52 ± 2.69
INH *^a^*	100 ± 1.86	100 ± 1.20	46.22 ± 2.13	30.81 ± 1.22	25.42 ± 3.84
Negative control	0 ± 1.20	2.53 ± 6.43	0 ± 2.86	1.77 ± 7.50	0 ± 2.29

*^a^* 2.92 μM.

**Table 4:** The cytotoxicity of the compounds in different cell lines.

Compound	Cell viability (200 μM) (%)	
	A549	THP-1
**1**	22.30 ± 0.19	66.58 ± 0.52
**5**	105.14 ± 7.39	104.69 ± 14.65
**6**	81.85 ± 0.32	67.14 ± 0.26
**7**	103.69 ± 3.44	76.57 ± 0.52
Negative control	101.71 ± 2.55	103.39 ± 1.83

## Supplementary Materials

The following are available online. Figures S1–S7: NMR spectra and HREIMS of **1**, Figures S8–S14: NMR spectra and HREIMS of **2**, Figures S15–S21: NMR spectra and HREIMS of **3**, Figures S22–S28: NMR spectra and HREIMS of **4**.
