# Supplementary material for: Anti-Mycobacterium tuberculosis Terpenoids from Resina Commiphora
Source: Molecules. 2019 Apr 15;24(8):1475. doi: 10.3390/molecules24081475 (PMC6515556; doi:10.3390/molecules24081475)
Supplement: Supplementary file 1 [file molecules-24-01475-s001.pdf]

Supplementary Materials

Anti-*Mycobacterium tuberculosis* Terpenoids from *Resina Commiphora*

Chuan-Zhi Zhu [1,2,†], Bin-Yuan Hu [2,†], Jia-Wang Liu [2,†], Yi Cai [2], Xin-Chun Chen [2], Da-Peng Qin [2], Yong-Xian Cheng [2,*] and Zong-De Zhang [1*]

1 Laboratory of Molecular Biology, Beijing Tuberculosis and Thoracic Tumor Research Institute, Beijing 101149, China; 15910404956@163.com;

2 Guangdong Key Laboratory for Genome Stability & Disease Prevention, School of Pharmaceutical Sciences, School of Basic Medicine, Shenzhen University Health Science Center, Shenzhen 518060, China; HuBinyuan2018@163.com (B.-Y.H.); liujiawang@imm.ac,cn (J.-W.L.); caiyi0113@gmail.com (Y.C.);chenxinchun@szu.edu.cn (X.-C.C.); tqindp@szu.edu.cn (D.-P.Q.)

* Correspondence: yxcheng@szu.edu.cn (Y.-X.C.); zzd417@163.com (Z.-D.Z.); Tel./Fax: +86-0755-26902073 (Y.-X.C.); +86-10-89509364 (Z.-D.Z.)

† These authors contributed equally to this work.

Received: 5 March 2019; Accepted: 12 April 2019; Published: 15 April 2019

Table of Contents

1. **Figures S1–S7 NMR spectra and HRESIMS of 1**
2. **Figures S8–S14 NMR spectra and HRESIMS of 2**
3. **Figures S15–S21 NMR spectra and HRESIMS of 2**
4. **Figures S2–S28 NMR spectra and HRESIMS of 2**

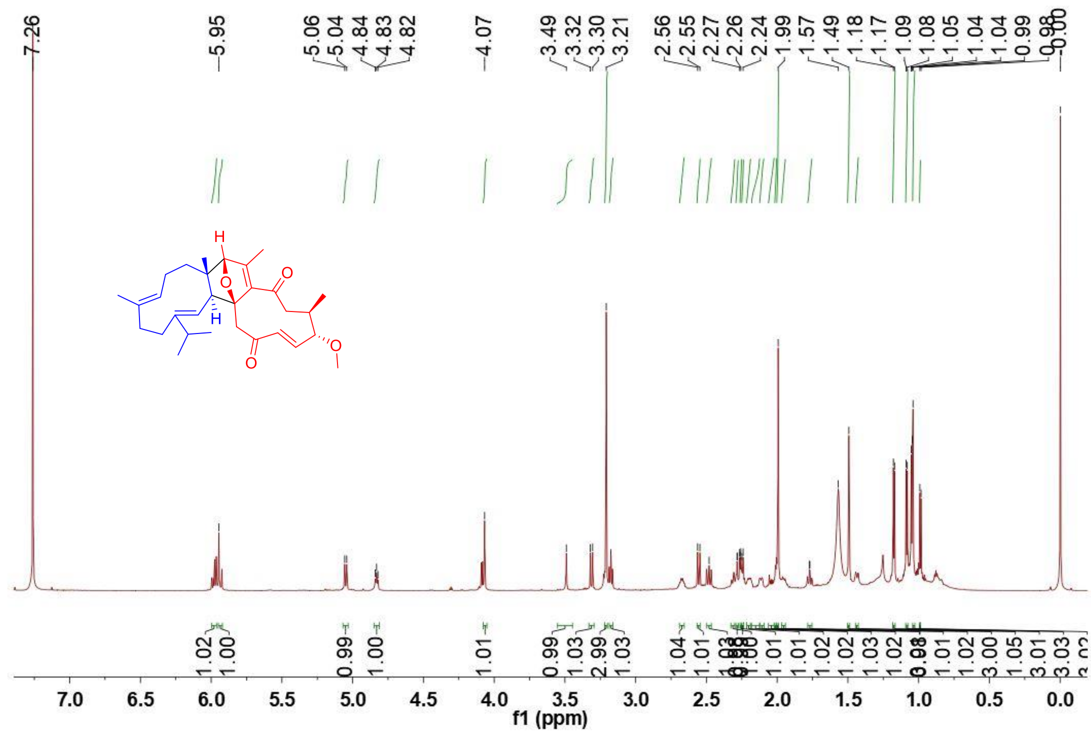

Figure S1.^{1}H NMR spectrum of **1** in CDCl3.

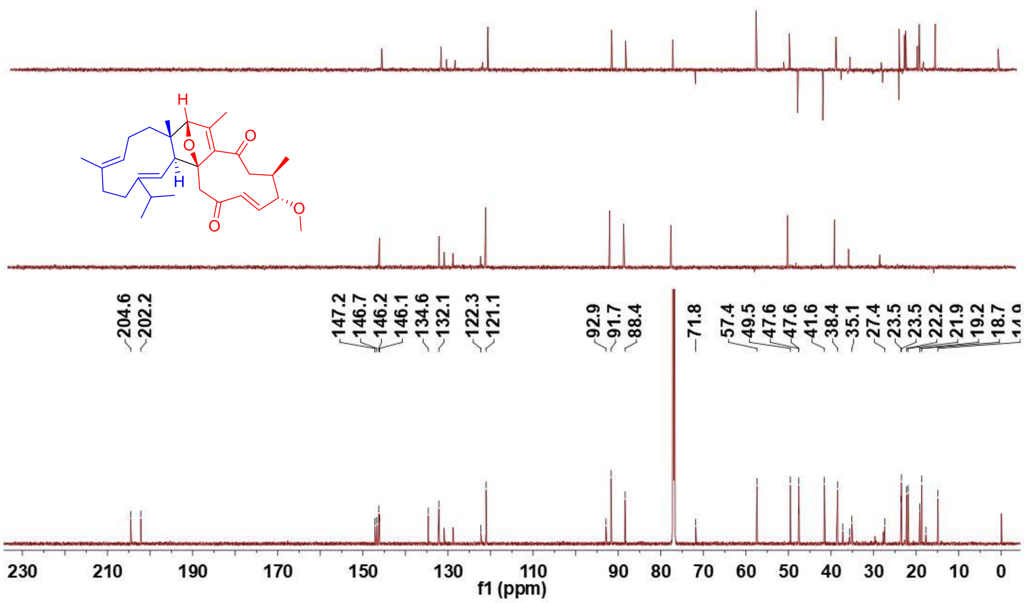

Figure S2. ^{13}C NMR and DEPT spectra of **1** in CDCl3.

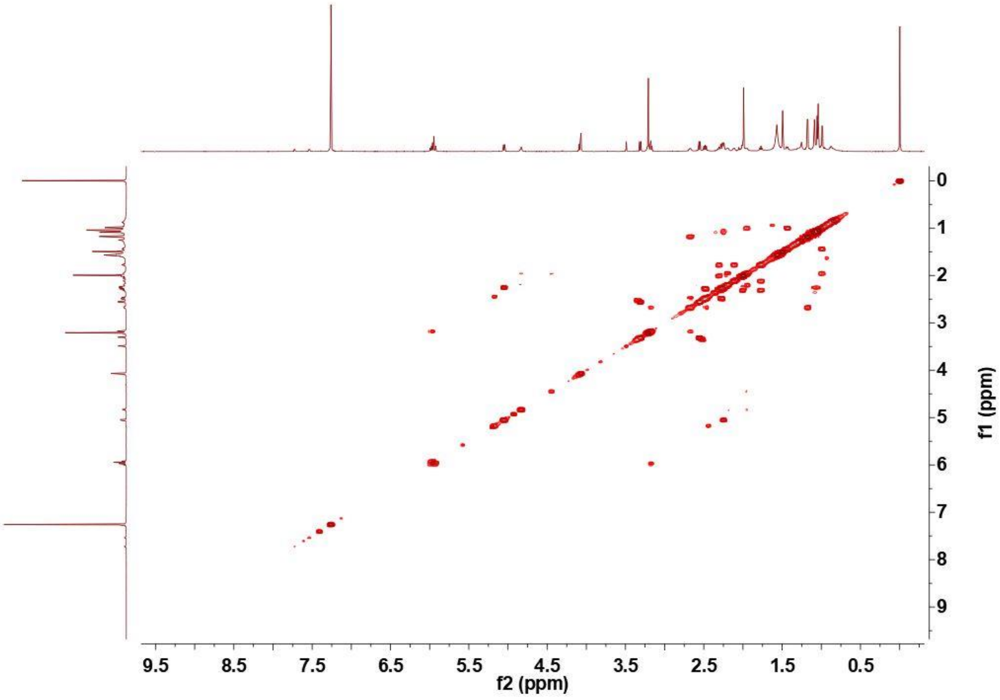

Figure S3. ^{1}H-^{1}H COSY spectrum of **1** in $CDCl_3$.

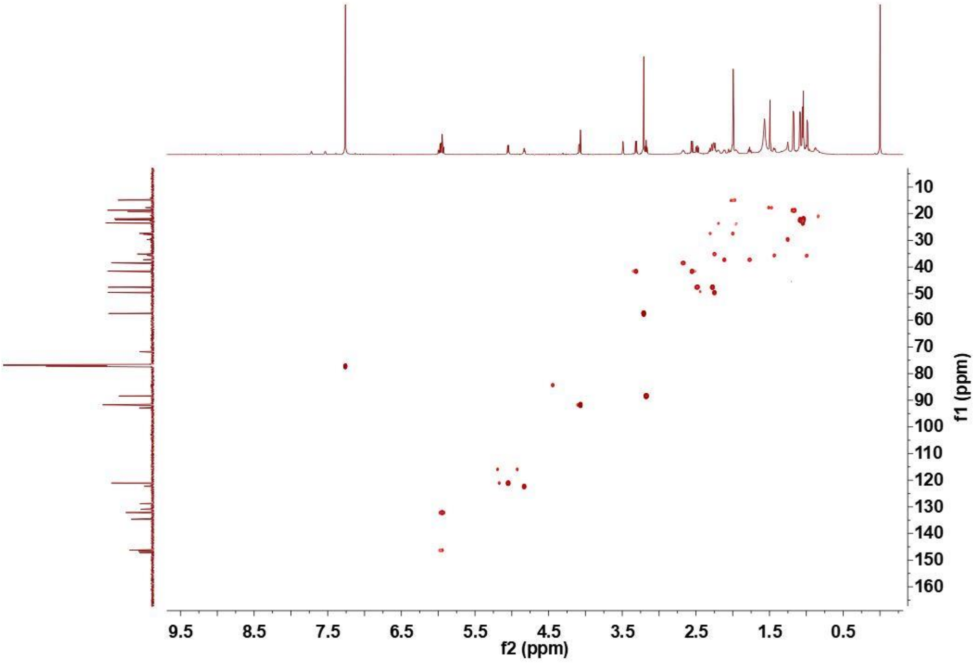

Figure S4. HSQC Spectrum of **1** in $CDCl_3$.

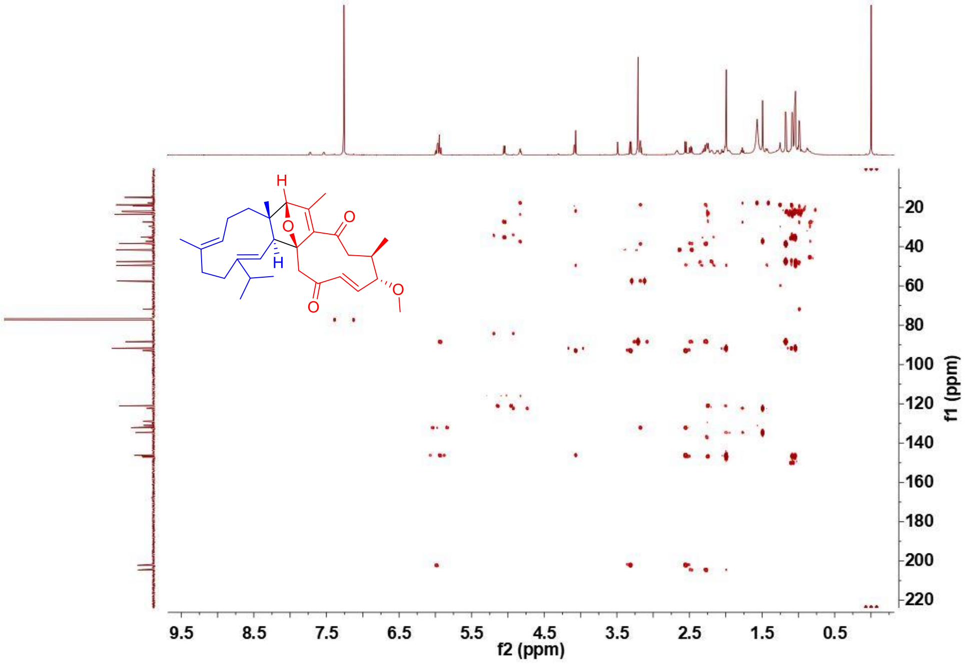

Figure S5. HMBC spectrum of **1** in $CDCl_3$.

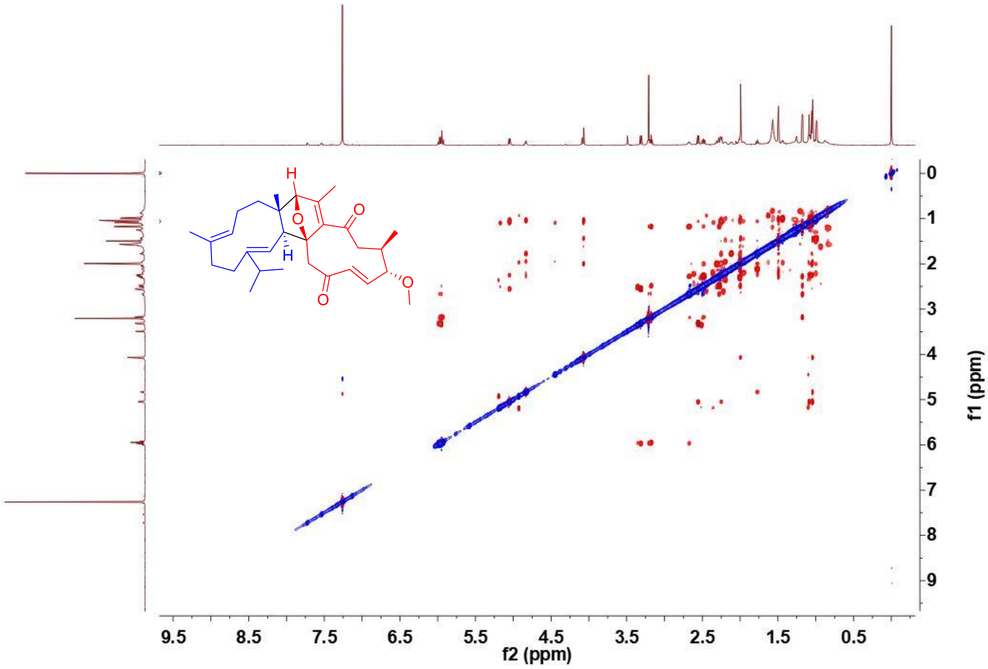

Figure S6. ROESY spectrum of **1** in $CDCl_3$.

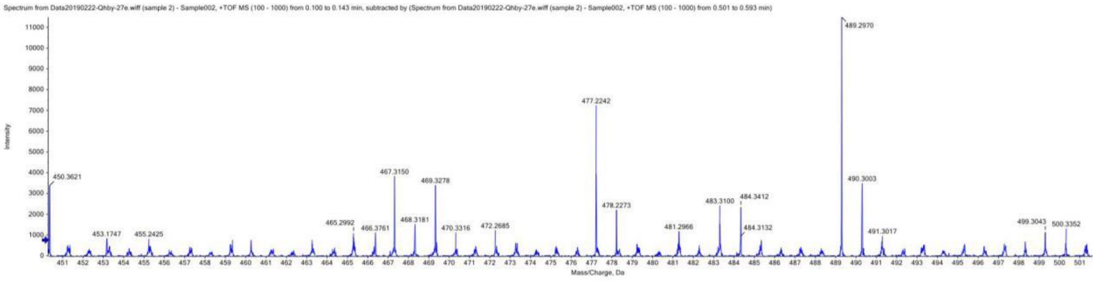

[M+H]+ m/z 467.3150

Hit	Formula	m/z	RDB	ppm
1	C30H43O4	467.3156	10.0	-1.3

Elements from ~ to C60H120O60

Mass tolerance 5 ppm

Figure S7. HRESIMS of **1**.

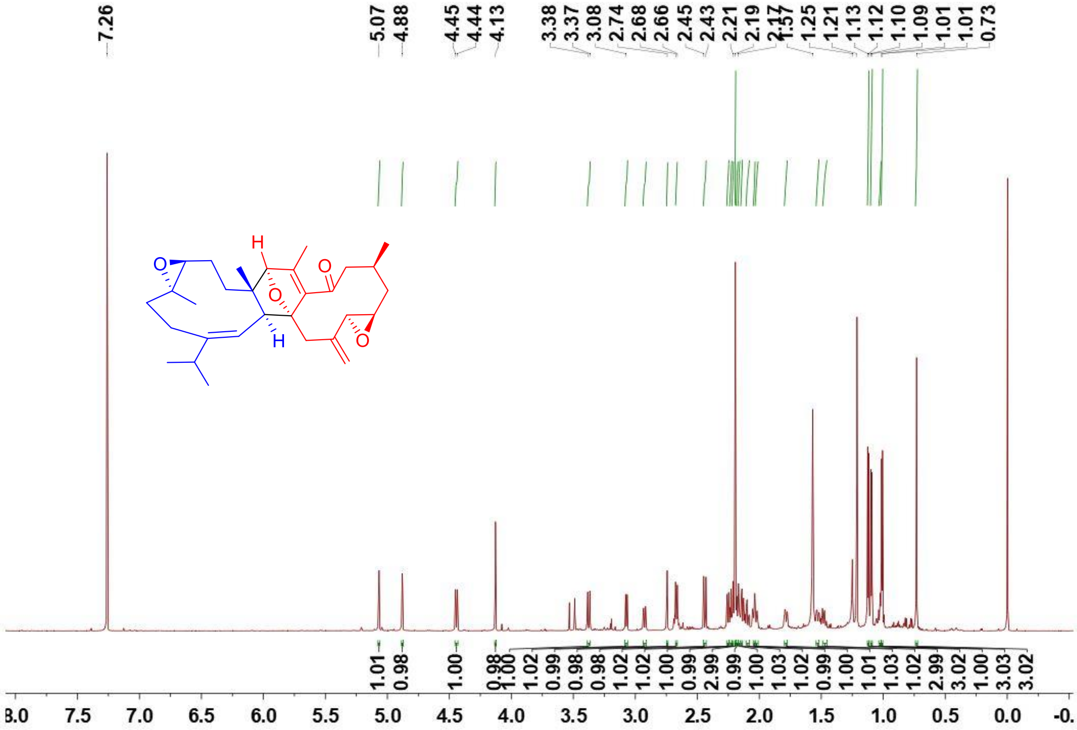

Figure S8. ^{1}H NMR spectrum of **2** in $CDCl_3$.

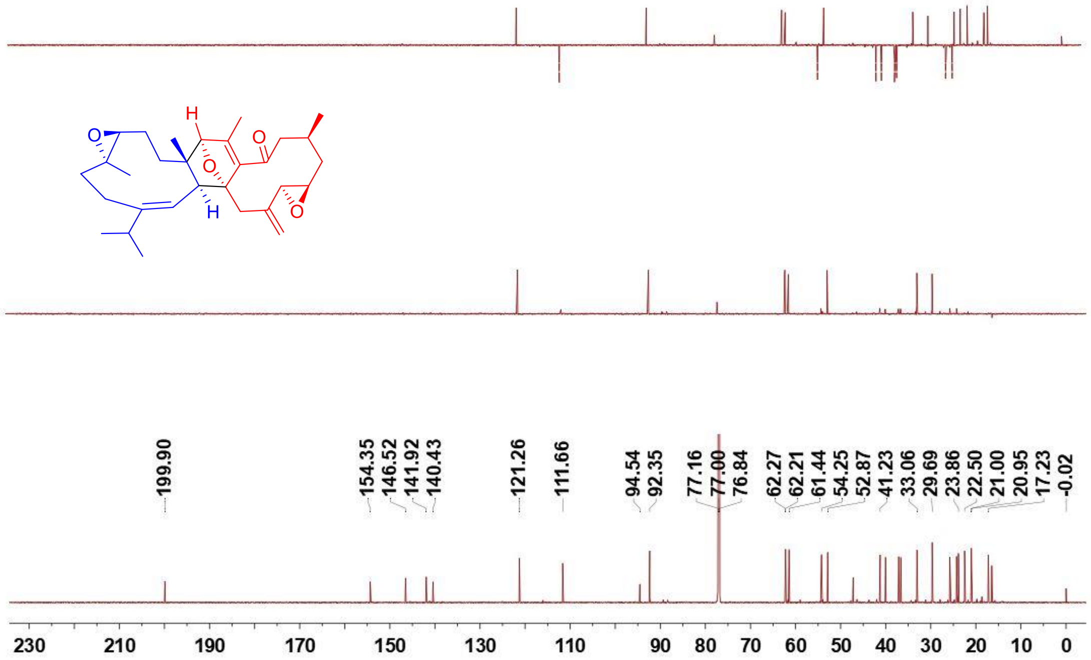

Figure S9. ^{13}C NMR and DEPT spectra of **2** in $CDCl_3$.

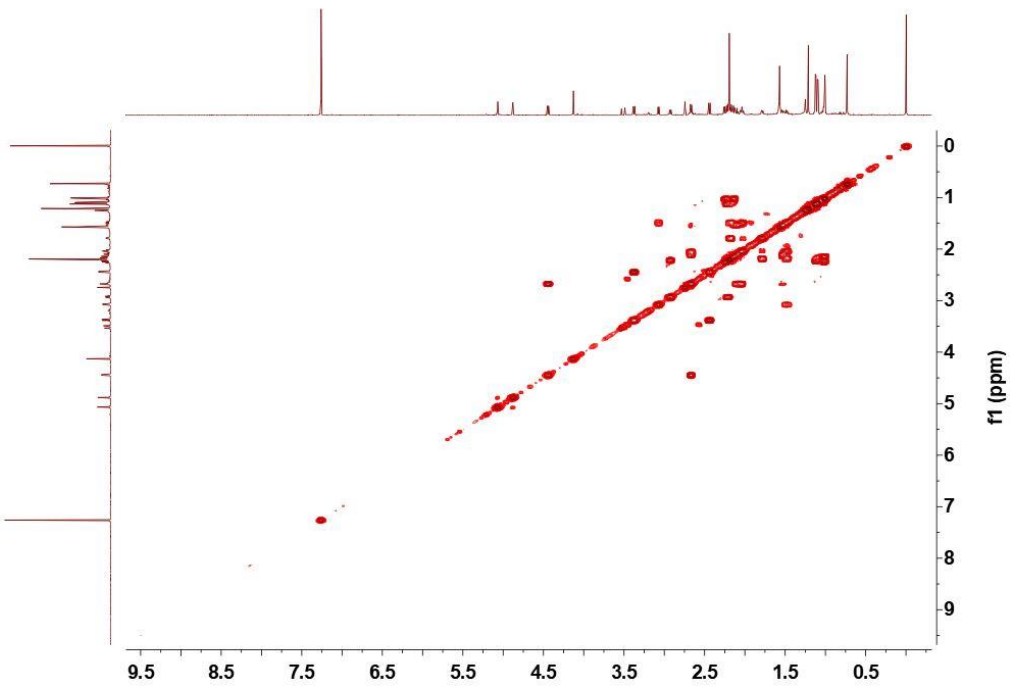

Figure S10. ^{1}H–^{1}H COSY spectrum of **2** in $CDCl_3$.

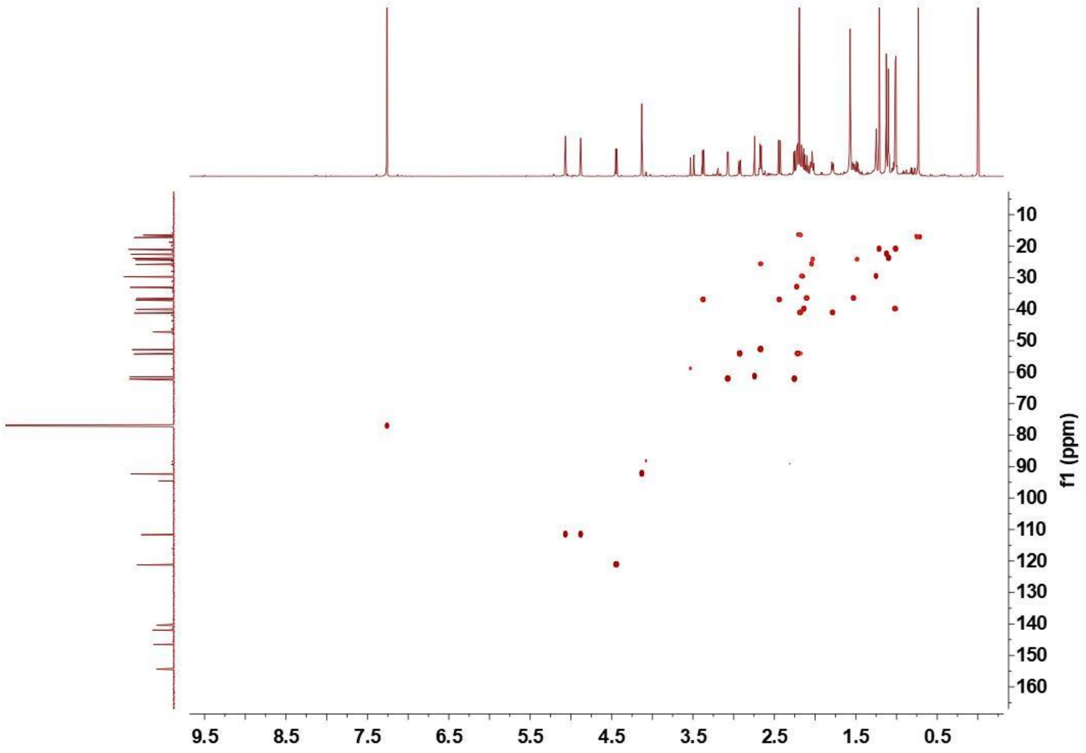

Figure S11. HSQC Spectrum of **2** in $CDCl_3$.

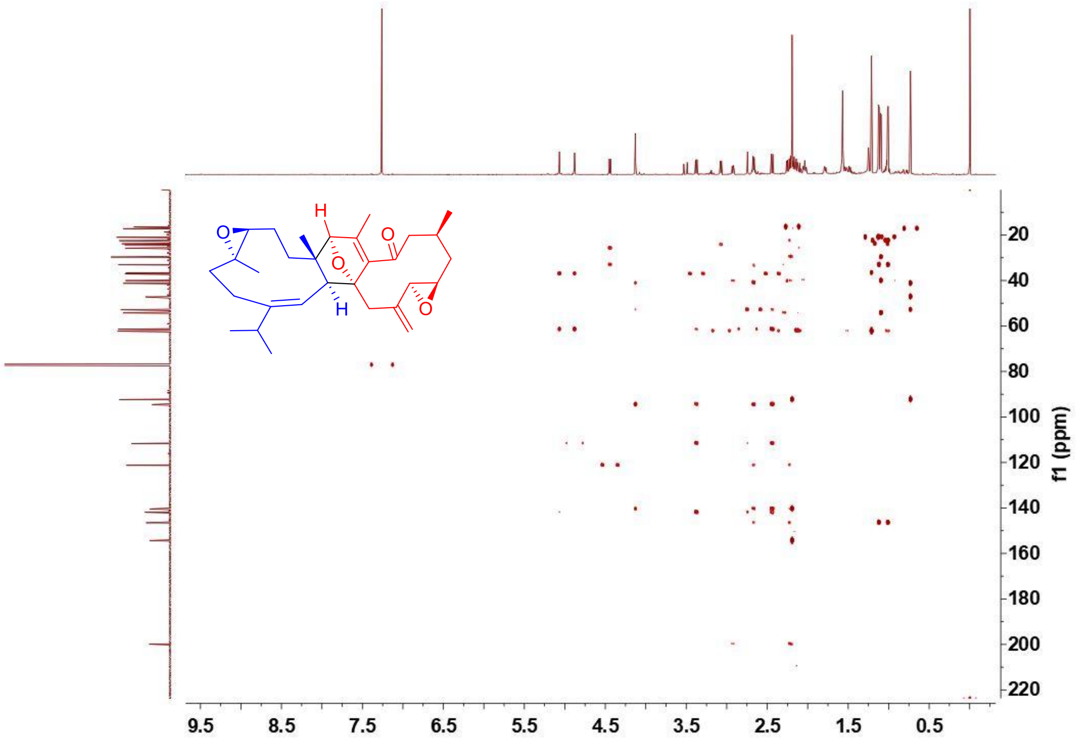

Figure S12. HMBC spectrum of **2** in $CDCl_3$.

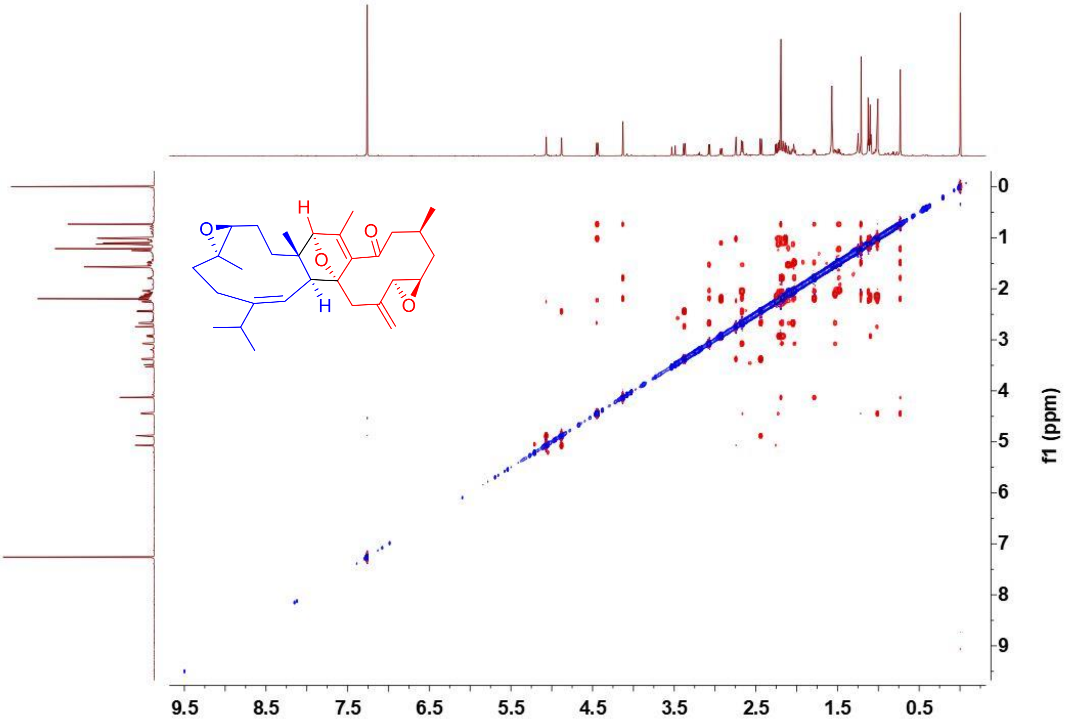

Figure S13. ROESY spectrum of **2** in $CDCl_3$.

Qualitative Analysis Report

**Data Filename**	170920ESIA15.d	**Sample Name**	qljw-58d
**Sample Type**	Sample	**Position**	
**Instrument Name**	Agilent G6230 TOF MS	**User Name**	KIB
**Acq Method**	ESI.m	**Acquired Time**	9/19/2017 6:28:58 PM
**IRM Calibration Status**	Success	**DA Method**	ESI.m
**Comment**			

**Sample Group**		**Info.**
**Acquisition SW**	6200 series TOF/6500 series	
**Version**	Q-TOF B.05.01 (B5125.2)	

User Spectra

Fragmentor Voltage	Collision Energy	Ionization Mode
250	0	ESI

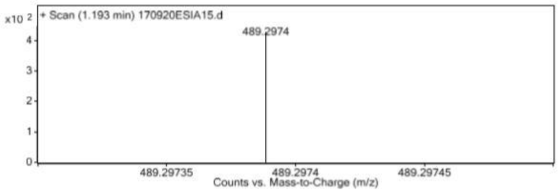

Peak List

m/z	z	Abund
121.0509	1	9492.1
256.2626	1	8474.39
274.2738	1	15405.78
318.2998	1	14130.2
362.3261	1	4403.11
384.3077	1	3165.42
437.1929	1	4847.29
521.3206	1	2700.71
922.0098	1	44952.27
923.01	1	5919.36

Formula Calculator Element Limits

Element	Min	Max
C	0	200
H	0	400
O	4	10
Na	1	1

Formula Calculator Results

Formula	CalculatedMass	Mz	Diff.(mDa)	Diff. (ppm)	DBE
C30 H42 Na O4	489.2981	489.2974	0.7	1.4	9.5

--- End Of Report ---

Figure S14. HRESIMS of **2.**

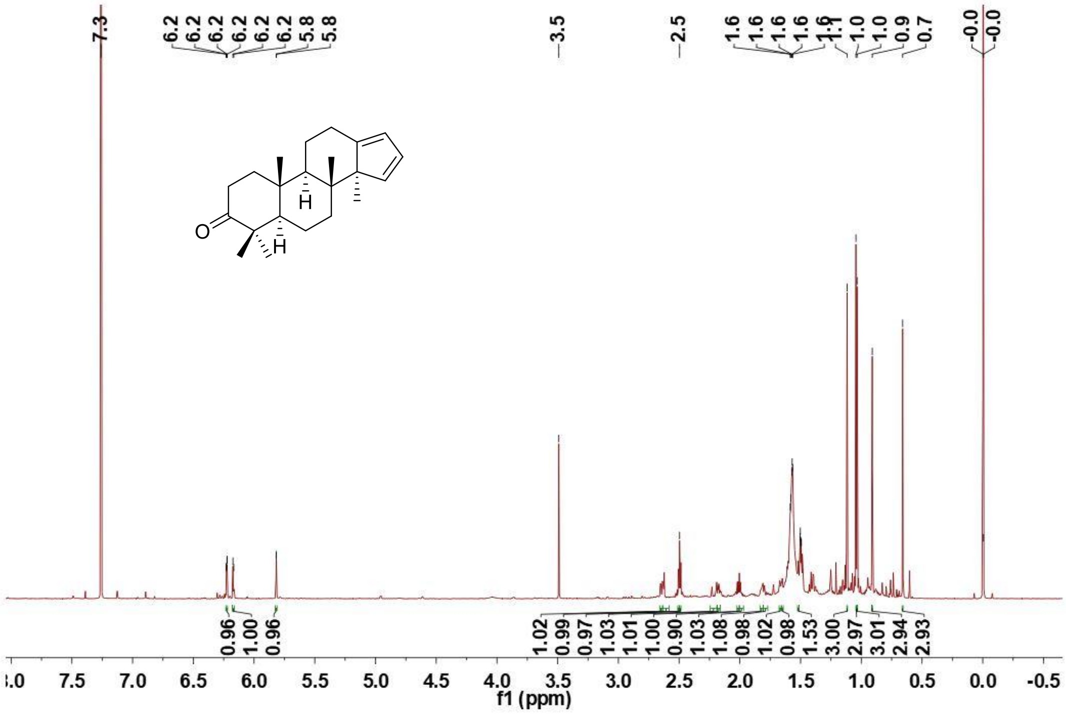

Figure Figure S15.^{1}H NMR spectrum of **3** in $CDCl_3$.

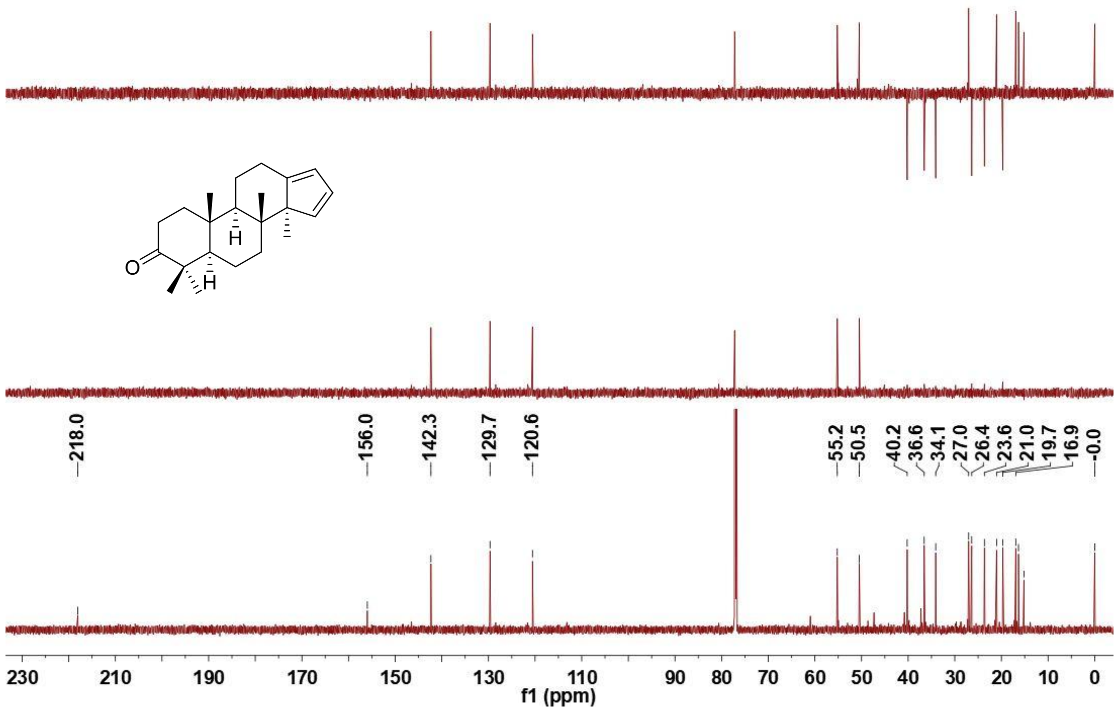

Figure S16. ^{13}C NMR and DEPT spectra of **3** in $CDCl_3$.

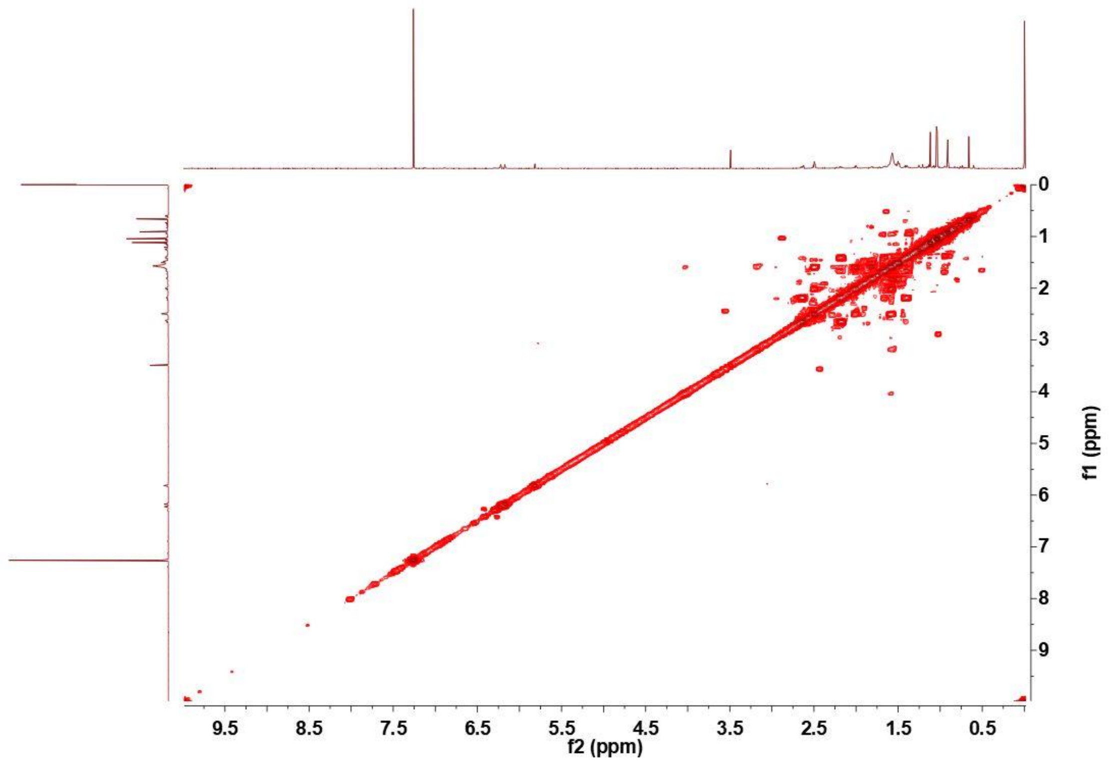

Figure S17. $^{1}H-^{1}H$ COSY spectrum of **3** in $CDCl_3$.

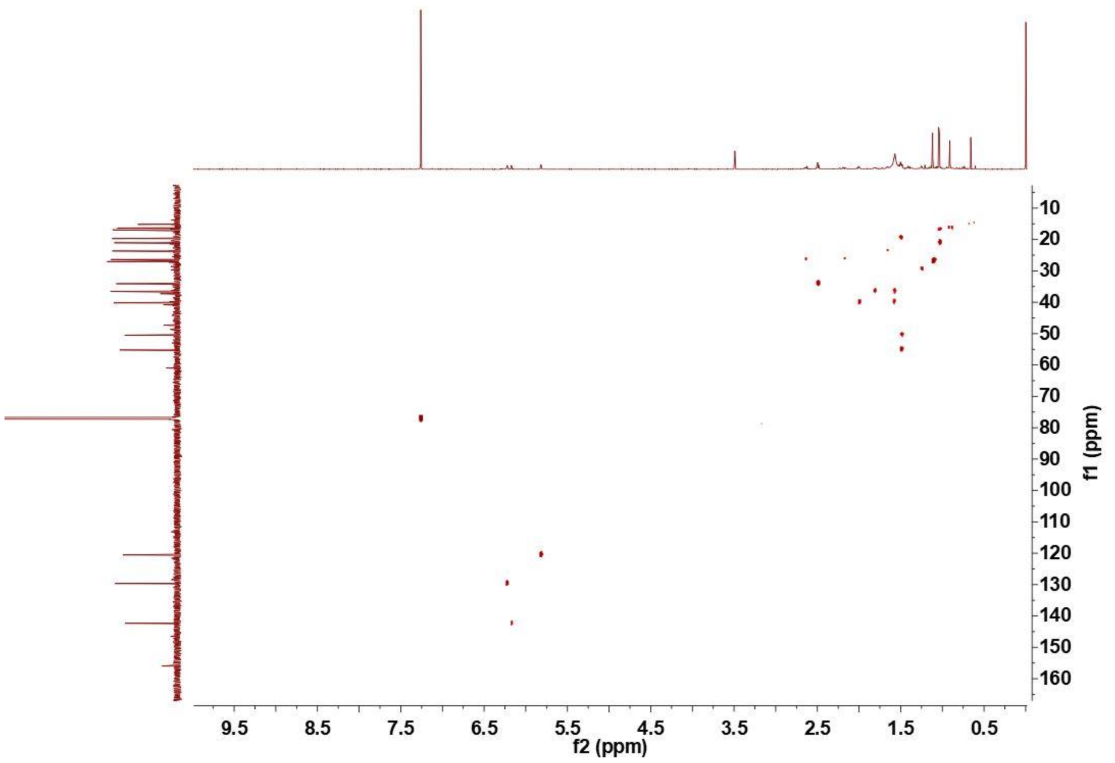

Figure S18. HSQC Spectrum of **3** in $CDCl_3$.

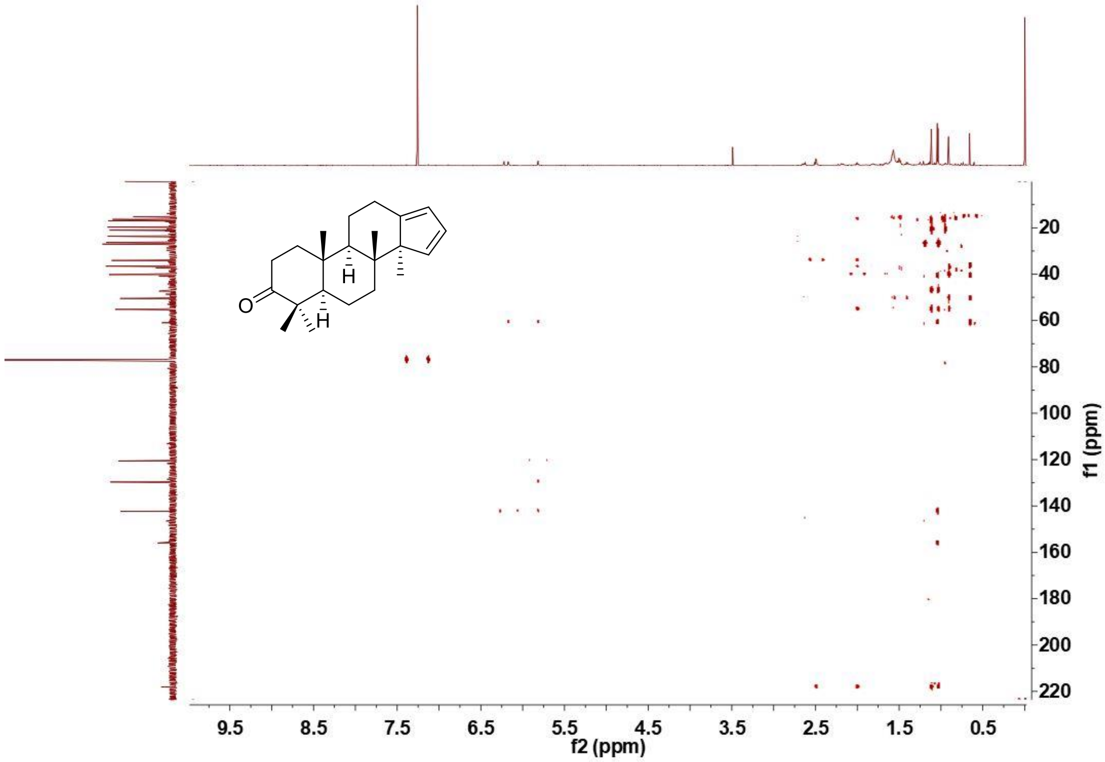

Figure S19. HMBC spectrum of **3** in $CDCl_3$.

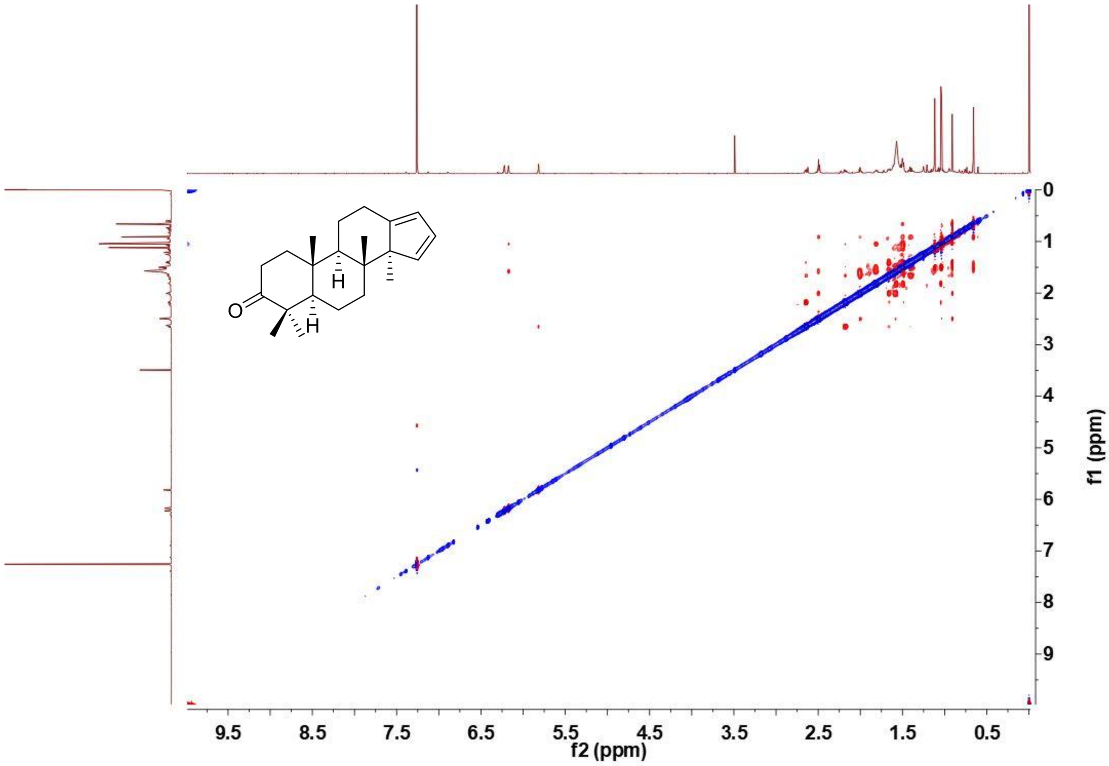

Figure S20. ROESY spectrum of **3** in $CDCl_3$.

Elemental Composition Report Page 1

Single Mass Analysis
Tolerance = 10.0 PPM / DBE: min = -10.0, max = 120.0
Selected filters: None

Monoisotopic Mass, Odd and Even Electron Ions
17 formula(e) evaluated with 1 results within limits (up to 51 closest results for each mass)
Elements Used:
C: 0-200 H: 0-400 O: 0-2

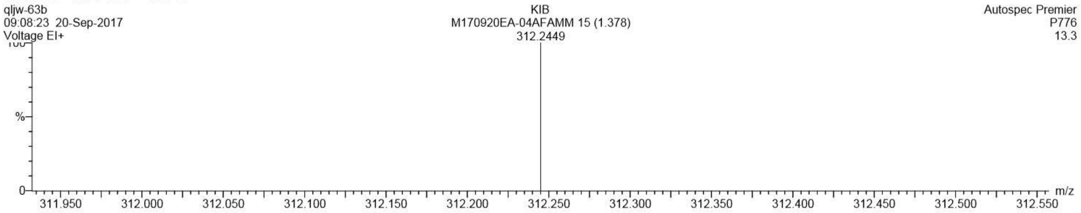

	Mass	Calc. Mass	mDa	PPM	DBE	i-FIT	Formula
Minimum:					-10.0		
Maximum:			200.0	10.0	120.0		
	312.2449	312.2453	-0.4	-1.3	7.0	5546026.5	C22 H32 O

Figure S21. HRESIMS of **3**

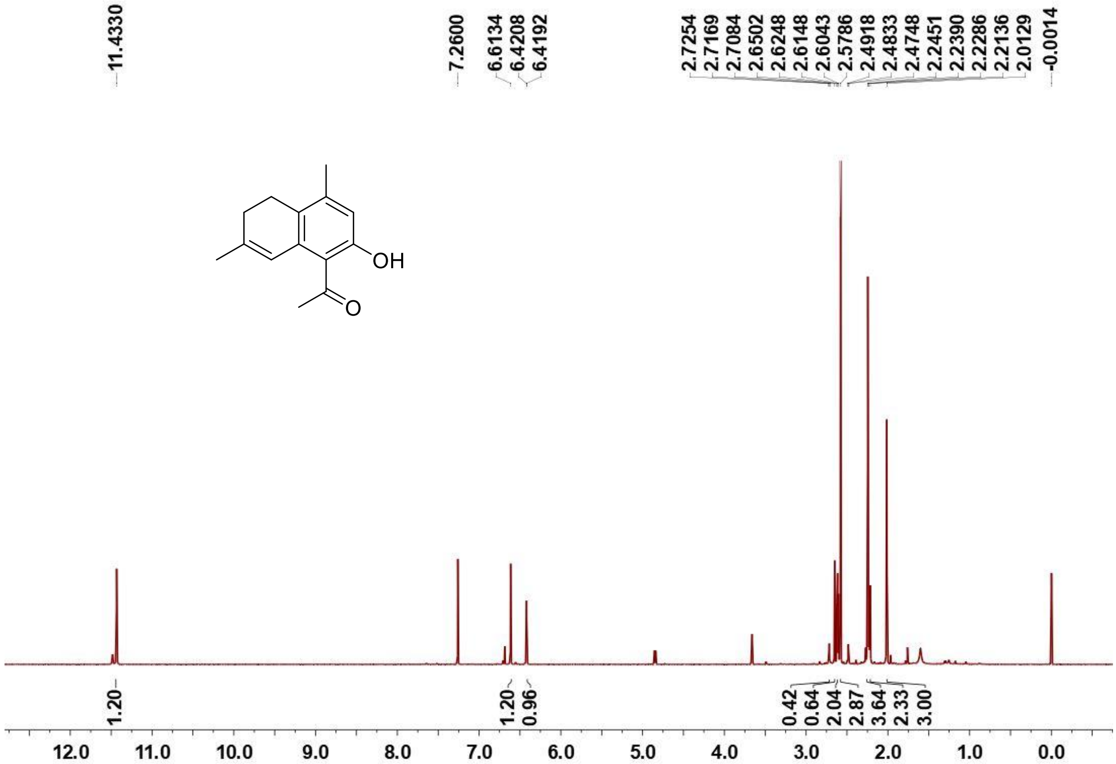

Figure Figure S22. ^{1}H NMR spectrum of **4** in $CDCl_3$.

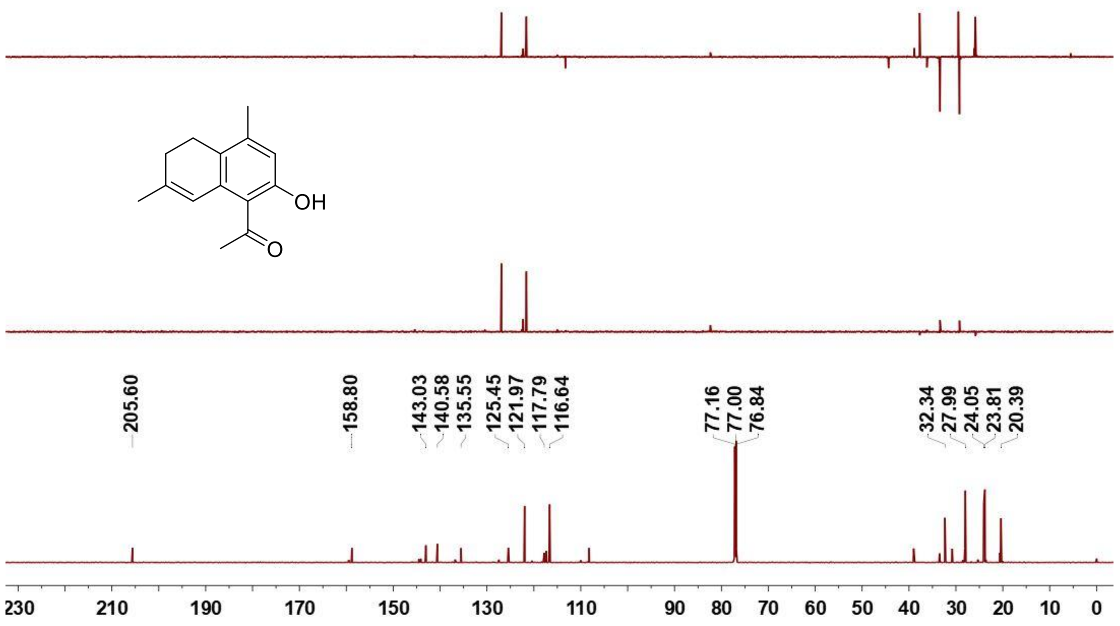

Figure S23. ^{13}C NMR and DEPT spectra of **4** in $CDCl_3$.

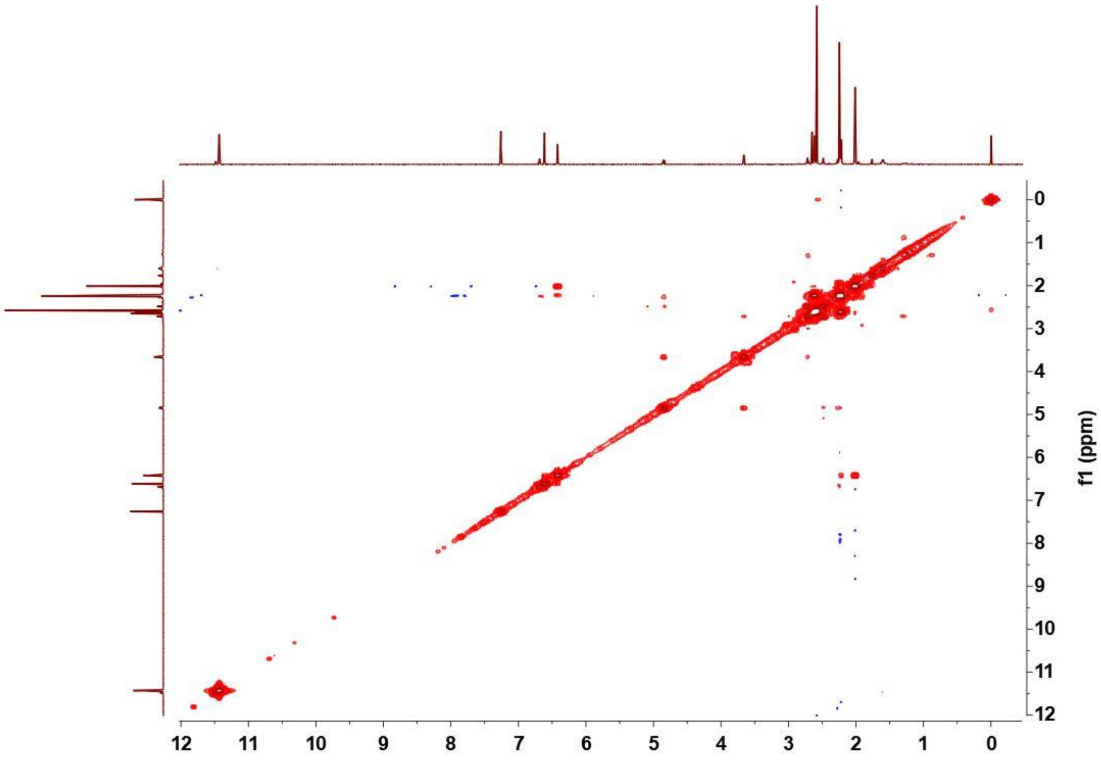

Figure S24. $^{1}H-^{1}H$ COSY spectrum of **4** in $CDCl_3$.

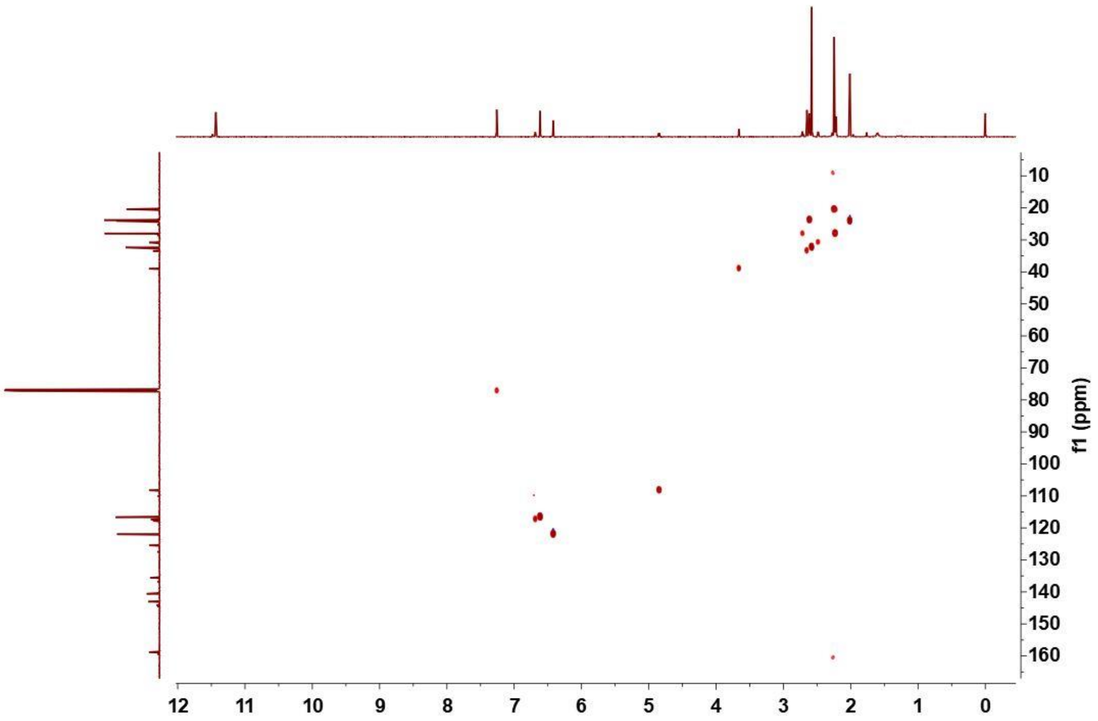

Figure S25. HSQC Spectrum of **4** in $CDCl_3$.

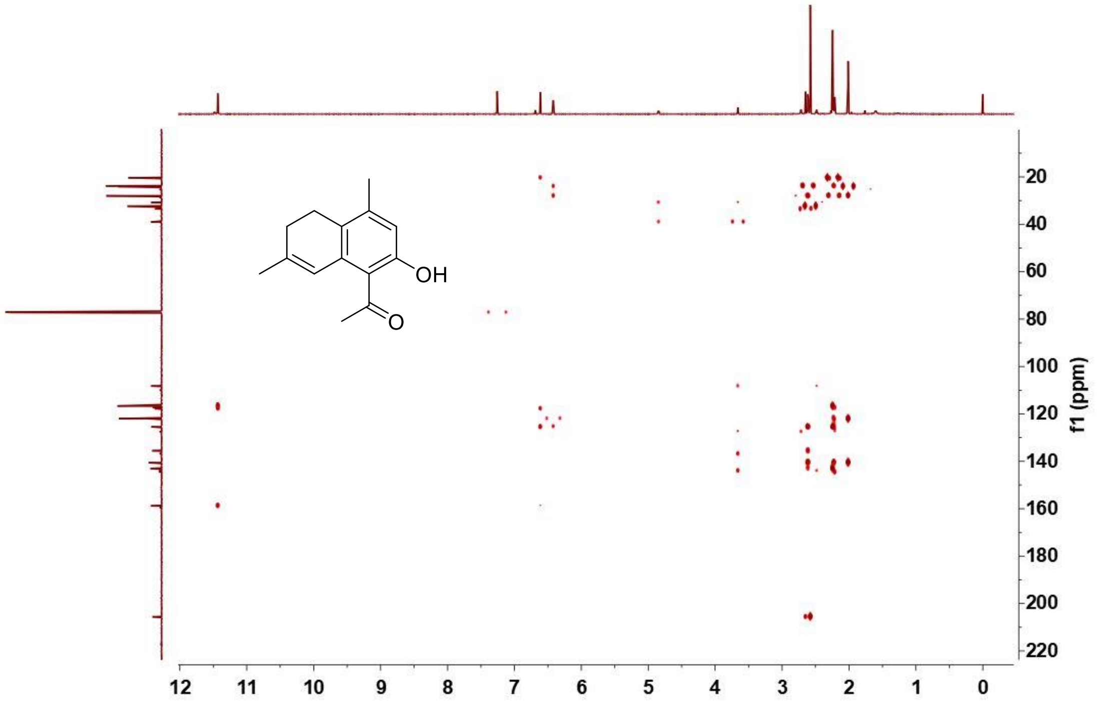

Figure S26. HMBC spectrum of **4** in $CDCl_3$.

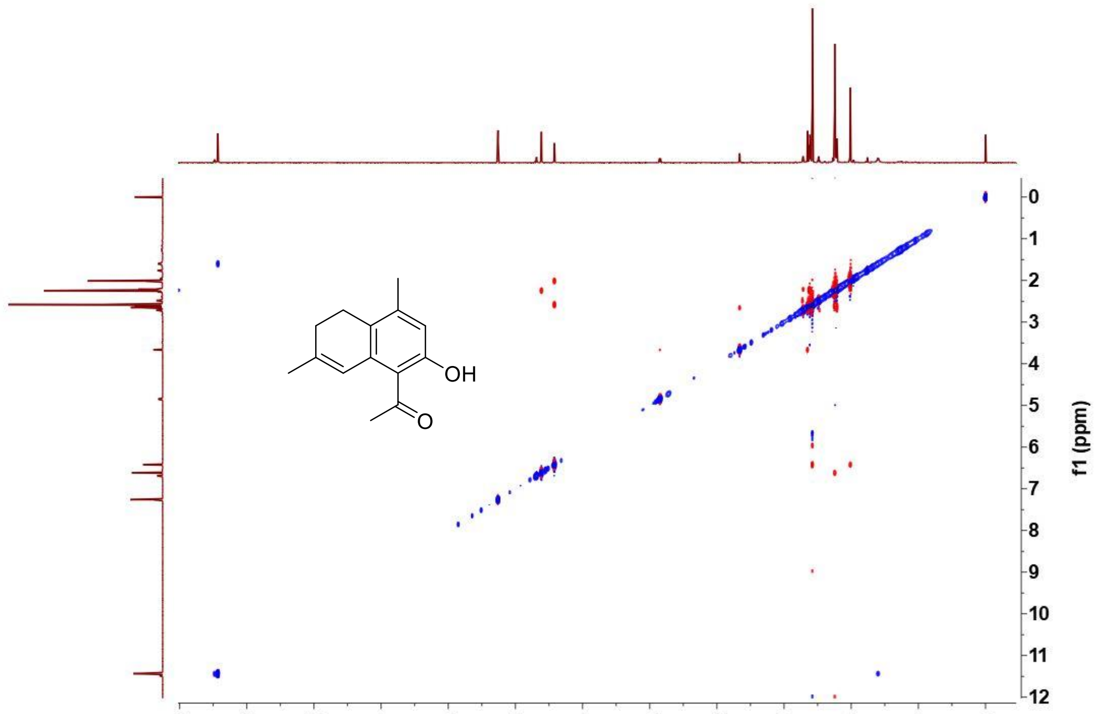

Figure S27. ROESY spectrum of **4** in $CDCl_3$.

Qualitative Analysis Report

**Data Filename**	qljw-31g.d	**Sample Name**	qljw-31g
**Sample Type**	Sample	**Position**	P1-A5
**Instrument Name**	Instrument 1	**User Name**	
**Acq Method**	SIBU.m	**Acquired Time**	5/15/2017 4:37:48 PM
**IRM Calibration Status**	Success	**DA Method**	Default.m
**Comment**			

Sample Group **Info.**
Acquisition SW 6200 series TOF/6500 series
Version Q-TOF B.05.01 (B5125.2)

User Spectra

Fragmentor Voltage	Collision Energy	Ionization Mode
150	0	ESI

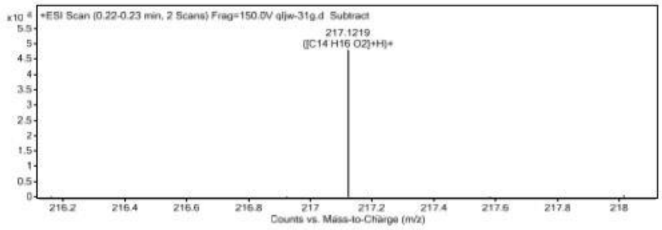

Peak List

m/z	z	Abund	Formula	Ion
128.0384	2	21256.65		
148.5519	2	7633.8		
217.1219	1	47814.5	C14 H16 O2	(M+H)+
227.1063		7368.16		
239.1041	1	40431.36		
255.0699	1	7581.44		
255.0965		7992.23		
267.0987	1	10794.19		

Formula Calculator Element Limits

Element	Min	Max
C	3	60
H	0	120
O	0	30

Formula Calculator Results

Formula	CalculatedMass	CalculatedMz	Mz	Diff. (mDa)	Diff. (ppm)	DBE
C14 H16 O2	216.1150	217.1223	217.1219	0.3	1.4	7.0000

--- End Of Report ---

Figure S28. HRESIMS of **4.**
